# Gut microbiota-derived tryptophan metabolites in cardiovascular disease: mechanisms, biomarkers, and precision interventions

**DOI:** 10.3389/fmicb.2026.1947695

**Published:** 2026-09-29

**Authors:** Qian Ding, Siqi Li, Mu Guo, Jia Pu, Jun Wei, Wei Chen

**Affiliations:** 1 Shanghai Fourth People's Hospital, School of Medicine, Tongji University, Shanghai, China; 2 Department of Cardiology, Shanghai Fourth People's Hospital, School of Medicine, Tongji University, Shanghai, China; 3 Leiden University Medical Center, Eindhoven Laboratory, Leiden University, Leiden, Netherlands; 4 Edinburgh Medical School: Biomedical Sciences, College of Medicine and Veterinary Medicine, University of Edinburgh, Edinburgh, United Kingdom

**Keywords:** aryl hydrocarbon receptor, cardiovascular disease, gut microbiota, indole derivatives, tryptophan metabolism

## Abstract

The gut microbiota plays a significant role in cardiovascular disease (CVD), through its metabolic products. Tryptophan is a key signaling molecule in the microbiota–host network; its gut microbial metabolites, including indole derivatives, kynurenine and serotonin, have dual effects on cardiovascular homeostasis. This review summarizes research progress on gut microbiota-derived tryptophan metabolites in atherosclerosis, hypertension, heart failure and other CVDs, focusing on metabolic flux in three pathways, the indole, kynurenine (KYN) and 5-hydroxytryptamine (5-HT) pathways, and their mechanisms via gut barrier maintenance, immune regulation through Th17/Treg and aryl hydrocarbon receptor (AhR) axis, and neuroendocrine blood pressure control. It also evaluates clinical evidence for key metabolites, such as indoxyl sulfate (IS), indole-3-propionic acid (IPA) and indole-3-acetic acid (IAA), as novel biomarkers, and explores precision interventions including dietary regimens, probiotic or postbiotic supplementation, traditional Chinese medicine ingredients, and host genetic background-guided strategies. Current evidence shows these metabolites exert both protective and pathogenic effects, highly dependent on disease context, microenvironment and host genetics. However, most data are from animal models and small cohort studies; remaining challenges include detection standardization, causality establishment and clinical translation. Future research should employ large prospective cohorts, multi-omics integration and mechanism-driven intervention trials to systematically elucidate the regulatory network and advance precision prevention and treatment.

## Graphical abstract

**Figure:**
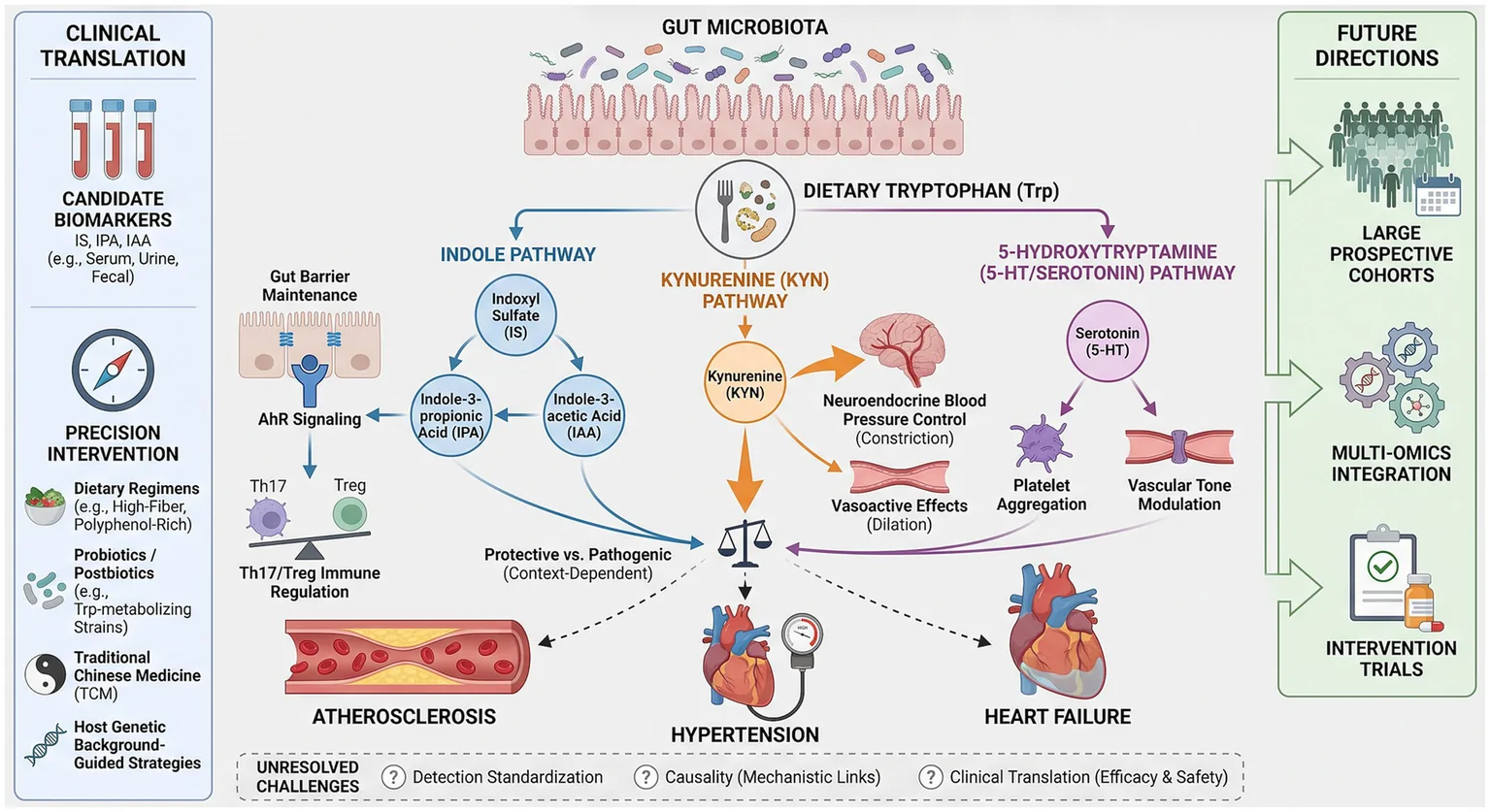

## 1 Introduction

In recent years, the gut microbiota—often referred to as the human “second genome”—has attracted increasing attention for its central role in regulating host metabolism, immunity and inflammatory response (Mukherjee et al., 2018). A wealth of research indicates that the gut microbiota forms a complex interactive network with the host via its metabolites, constituting the “gut microbiota–host metabolic axis”, which plays a key regulatory role in the progression of cardiovascular diseases (CVD), such as atherosclerosis, hypertension and heart failure (Mukherjee et al., 2018; Wang and Zhao, 2018). Dysbiosis of the gut microbiota can lead to abnormalities in the metabolite profile, thereby influencing systemic inflammatory states, endothelial function, blood pressure regulation and the progression of atherosclerosis (Olić et al., 2026). For example, in an Ldlr^−^/^−^ mouse model, disrupted intestinal tryptophan (Trp) metabolism exacerbates aortic atherosclerotic lesions and is closely associated with increased intestinal permeability, endotoxemia and systemic inflammation (Delk et al., 2025). Furthermore, clinical studies have also found that levels of various microbiota-derived metabolites (such as butyrate) are significantly reduced in the plasma of patients with CVD, suggesting their potential protective role (Liao et al., 2021). More importantly, a multi-omics study of patients with intracranial aneurysms revealed that plasma levels of indoxyl sulfate (IS), derived from the gut microbiota, were significantly elevated in patients at the time of rupture; furthermore, a machine learning model based on this metabolite could efficiently distinguish between ruptured and unruptured aneurysms (area under the curve, AUC = 0.97). Animal experiments confirmed that depletion of the gut microbiota reduces IS levels and decreases aneurysm formation and rupture (Sun et al., 2024). Taken together, this evidence suggests that the gut microbiota profoundly influences the homeostasis of the cardiovascular system through its metabolic output; its dysbiosis is not only a risk factor for CVD but may also be directly involved in the pathophysiological mechanisms of the disease.

Tryptophan, as an essential amino acid for the human body, is not only utilized for protein synthesis but can also be metabolized by host enzyme systems or gut microbiota to produce various bioactive molecules. Among these, the gut microbiota-mediated Trp metabolic pathway has in recent years been demonstrated to be a crucial link between the gut microbiome and systemic health (Levé et al., 2025). Under healthy conditions, the commensal microbiota can convert Trp into indole and its derivatives (such as indole-3-lactic acid, indole-3-propionic acid and indole-3-acrylic acid). These metabolites not only contribute to maintaining the integrity of the intestinal barrier but also regulate immune homeostasis by activating signaling pathways such as the aryl hydrocarbon receptor (AhR) (Su et al., 2022; Paeslack et al., 2022). However, under conditions of dysbiosis or dietary changes (such as a high-fat, high-cholesterol diet), the direction of Trp metabolism may shift, leading to an imbalance in the ratio of harmful to protective metabolites, thereby affecting cardiovascular function (Mukherjee et al., 2018; Olić et al., 2026). For example, indole-3-propionic acid (IPA) exhibits a dual role in hypertension models: on the one hand, it promotes sodium excretion and lowers blood pressure by regulating the Th17/Treg balance within the kidneys (Baranwal et al., 2023); however, under certain experimental conditions, specifically, at suprapharmacological doses administered intravenously, IPA has also been reported to exert vasoconstrictive and transient pressor effects (Konopelski et al., 2021), suggesting that its action is highly context-dependent and influenced by administration route and dosage. Furthermore, metabolites such as kynurenine (KYN), serotonin and IS have also been shown to participate in the progression of various cardiovascular diseases, including atherosclerosis and heart failure, by regulating inflammation, oxidative stress and endothelial function (Liu et al., 2021; Li C. et al., 2024). Despite substantial preclinical and clinical evidence supporting the pivotal role of gut microbiota-derived Trp metabolites in cardiovascular disease (CVD), a systematic evidence-grading framework that stratifies the strength of findings from animal models to clinical cohorts remains absent. Additionally, the upstream determinants of metabolic flux allocation among the indole, kynurenine, and serotonin (5-HT) pathways, as well as the comprehensive landscape of intervention strategies—particularly those that incorporate traditional Chinese medicine ingredients and host genetic background-guided precision approaches—are still inadequately explored (Liu et al., 2022).

Accordingly, this review summarizes research progress on gut microbiota-derived Trp metabolites in cardiovascular disease, focusing on their molecular mechanisms, disease-specific associations and potential for targeted intervention, with a view to providing new perspectives for the precision prevention and treatment of CVD.

## 2 Literature search strategy and evidence grading

### 2.1 Literature search strategy

This paper is a narrative review, aiming to systematically summarize research progress on gut microbiota-derived Trp metabolites in cardiovascular disease, rather than adopting the methodological framework of a systematic review or meta-analysis. The literature search covered the following databases: PubMed and Web of Science. The search time frame spanned from the inception of each database to June 2026. The search query was: (tryptophan[Title/Abstract] OR indole[Title/Abstract] OR kynurenine[Title/Abstract] OR indoxyl sulfate[Title/Abstract] OR indole-3-propionic acid[Title/Abstract] OR indole-3-acetic acid[Title/Abstract] OR serotonin[Title/Abstract] OR 5-hydroxytryptamine[Title/Abstract] OR aryl hydrocarbon receptor[Title/Abstract] OR AhR[Title/Abstract]) AND (gut microbiota[Title/Abstract] OR microbiome[Title/Abstract] OR gut microbe[Title/Abstract]) AND (cardiovascular[Title/Abstract] OR atherosclerosis[Title/Abstract] OR hypertension[Title/Abstract] OR heart failure[Title/Abstract] OR coronary artery disease[Title/Abstract] OR myocardial infarction[Title/Abstract] OR aortic dissection[Title/Abstract] OR intracranial aneurysm[Title/Abstract]). Inclusion criteria were as follows: (1) original research (including animal studies, cell-based studies and clinical trials) and reviews published in peer-reviewed academic journals; (2) studies investigating the mechanisms of action, biomarker value or intervention strategies of Trp metabolites mediated by the gut microbiota in the cardiovascular system. Exclusion criteria were as follows: (1) conference abstracts, theses and preprints; (2) non-English language literature; (3) studies not directly relevant to the topic.

### 2.2 Methodology for evidence grading

To enable readers to systematically assess the relative strength of evidence from different sources, this paper adopts a modified version of the GRADE principles (suitable for reviews of biological mechanisms), classifying evidence into three levels: (1) Causal evidence: Studies derived from randomized controlled trials (RCTs) with clinical hard endpoints (e.g., cardiovascular events, mortality) as primary outcomes; or prospective cohort studies demonstrating significant associations after multivariable adjustment with a clear temporal sequence, with effect sizes and confidence intervals reported; Mendelian randomization (MR) studies must provide sensitivity analyses (e.g., MR-Egger, Cochran’s Q test) to verify the absence of horizontal pleiotropy in instrumental variables. (2) Correlation evidence: Cross-sectional studies, case–control studies, and RCTs or prospective studies with surrogate endpoints (e.g., metabolite levels, biomarkers) as primary outcomes reporting associations. (3) Mechanistic suggestive evidence: Mechanistic hypotheses provided by animal experiments, cell experiments, ex vivo tissue experiments, etc., with model limitations noted. The “Anecdotal evidence” level is not defined in the methodology; all animal or cell experiments are classified as mechanistic suggestive evidence (Table 1).

**Table 1:** Evidence grading of gut microbiota-derived tryptophan metabolites in cardiovascular diseases.

Diseases/Areas covered	Key arguments	Key metabolites / bacterial strains	Primary microbial sources	Study design/Model	Sample size	Level of evidence	Ref.
Atherosclerosis	Induces endothelial dysfunction and vascular inflammation by activating oxidative stress and inhibiting the NO/cGMP pathway	Indoxyl sulfate (IS)	*Escherichia coli, Bacteroides* spp.	*In vivo* + *in vitro* mechanistic study	Male Wistar rats (3–4 months), ex vivo thoracic aortic rings; *n* = 5–10 per group across experiments (ET-1 curves *n* = 9–10; antagonist/inhibitor assays *n* = 6–8; high-K^+^ and SOD activity *n* = 5–7)	Animal study + associative evidence (human data)	Matsumoto et al. (2021)
Atherosclerosis	Defects in the PSRC1 gene lead to reduced IAA levels via depletion of Akkermansia, impairing AhR activation and exacerbating plaque formation	Indole-3-acetic acid (IAA)	*Akkermansia muciniphila*	Animal intervention + human validation	Animals mice/cells *n* = 6–10/group (baseline *n* = 6; intervention *n* = 10, atorvastatin control *n* = 6; molecular *n* = 3–6); IAA toxicity experiments not reported; macrophages *n* = 3–6. Cohort *n* = 103 (CAD 77 vs. control 26)	Animal study + associative evidence (human data)	Wu et al. (2026)
Atherosclerosis	*Bacteroides ovatus* suppresses M1 and promotes M2 macrophage polarization by producing IAA, alleviating aortic inflammation	Indole-3-acetic acid (IAA)	*Bacteroides ovatus*	Population multi-omics + FMT + strain/metabolite intervention + cellular mechanisms	Animals (mice/cells) Mice *n* = 6/group (body weight *n* = 6–10; RNA-seq *n* = 3; WB *n* = 5); RAW264.7 cells WB *n* = 5, IF *n* = 3 independent experiments Cohort (human validation) *n* = 60 (ASCVD 30 vs. risk factor controls 30), 16S and serum IAA detection *n* = 30/group.	Animal study + associative evidence (multi-omics)	Liu et al. (2025)
Atherosclerosis (thrombosis)	Elevates circulating L-tryptophan levels via gut commensal, inducing platelet hyperreactivity and thrombosis	L-tryptophan	*Bacteroides thetaiotaomicron*	Population multi-omics cohort + germ-free mouse mechanistic validation	Animals: GF ApoE^−^/^−^ male mice — *B. thetaiotaomicron* vs. *P. copri* colonization for 3 weeks, fecal L-tryptophan *n* = 11/group; FeCl₃ carotid artery injury thrombosis model *n* = 8 (B.t) vs. 7 (P.c); platelet adhesion and flow-activated assays n not explicitly reported. Human: *n* = 146 (healthy controls 36 / stable CAD 64 / myocardial infarction 46), fecal metagenomics + serum metabolomics.	Animal study + associative evidence (human metagenomics)	Liu et al. (2024)
Atherosclerosis	Promotes anti-atherosclerotic effects by downregulating miR-142-5p and upregulating ABCA1 to facilitate cholesterol efflux from macrophages	Indole-3-propionic acid (IPA)	*Clostridium sporogenes, Peptostreptococcus* spp.*, Ruminococcus* spp.	Prospective cohort multi-omics + germ-free mouse mechanistic validation with comparator strain	Animals: germ-free ApoE^−^/^−^ mice *n* = 11/group (fecal L-tryptophan); carotid thrombosis *n* = 8 vs. 7. Human: *n* = 146 (HC 36 / SCAD 64 / MI 46)	Animal study + associative evidence (independent cohort)	Xue et al. (2022)
Atherosclerosis	The KYN/Trp ratio is independently associated with postoperative cardiovascular complications and long-term MACE in advanced-stage patients	KYN/Trp ratio	N/A	Pilot case–control targeted metabolomics (plasma HPLC–MS/MS)	Human: *n* = 122 (advanced atherosclerosis 100 vs. age/sex-matched controls 22). Postoperative outcomes: 30-day major cardiac complication *n* = 14 (14%); follow-up MACE *n* = 20 (20%)	Associative evidence	Cason et al. (2018)
Hypertension	Promotes sodium excretion and lowers blood pressure by modulating the renal Th17/Treg immune balance	Indole-3-propionic acid (IPA)	*Clostridium sporogenes, Peptostreptococcus* spp.*, Ruminococcus* spp.	Animal intervention + in vitro validation	Animals: C57BL/6 J mice *n* = 4–6/group (serum/fecal IPA *n* = 6; IPA supplementation 4 groups × *n* = 6; SBP *n* = 4/group); in vitro T-cell differentiation *n* = 6/group	Mechanism-suggestive evidence	Baranwal et al. (2023)
Hypertension	Exhibits dose-dependent vasoconstriction and increased blood pressure in isolated vessels and whole animals, contradicting the above effects	Indole-3-propionic acid (IPA)	*Clostridium sporogenes, Peptostreptococcus* spp.*, Ruminococcus* spp.	In vivo hemodynamic + ex vivo vascular + in vitro cell study	Animals: rats *n* = 5–8/group (BP/ECG: vehicle *n* = 6, IPA 1.6/53 μM/kg *n* = 6, 5.3 μM/kg *n* = 8; prazosin/pentolinium *n* = 5 each; ECHO *n* = 6/group; plasma IPA kinetics *n* = 5); mesenteric arteries 24 from 14 rats; human cardiomyocytes 3 independent series × triplicates.	Mechanism-suggestive evidence	Konopelski et al. (2021)
Hypertension	In obese adolescents, IPA is negatively correlated with diastolic BP and lipids, while IS is positively correlated with fasting glucose	IPA / IS	*IPA: Clostridium sporogenes; IS: E. coli, Bacteroides* spp.	Cross-sectional human study	Human: *n* = 28 adolescents with obesity (13–18 y; mean BMI 36 ± 6.4 kg/m^2^; class I *n* = 12 / II *n* = 7 / III *n* = 9; 54% boys; Hispanic-White *n* = 14 / non-Hispanic Black *n* = 11 / non-Hispanic White *n* = 3)	Associative evidence	Rivera et al. (2025)
Heart failure	Disrupts mitochondrial function by activating the AhR-CYP1B1 axis in cardiomyocytes, driving CKD-associated heart failure	Indoxyl sulfate (IS)	*E. coli, Bacteroides* spp.	In vivo + in vitro mechanistic study	Animals: male SD rats *n* = 6/group (Sham vs. 5/6-nephrectomy UCM, 12-week model)	Mechanism-suggestive evidence	Lu et al. (2024)
Heart failure	Mendelian randomization supports a causal association between gut microbiota–derived metabolites and heart failure; tryptophan metabolites may serve as mediators	Circulating tryptophan metabolites	*Various gut microbial species* (*indirectly via metabolite production*)	Mendelian randomization (MR)	GWAS summary-data MR. Exposures: gut microbiota *n* = 5,959 (FINRISK 2002, 473 taxa); circulating metabolites *n* = 136,016 (33 cohorts, 233 metabolites). Outcomes: CAD 122,733 cases vs. 424,528 controls; T2DM 12,931 vs. 57,196; stroke 40,585 vs. 406,111 (MEGASTROKE); heart failure 47,309 vs. 930,014; hypertension n not reported (MRC-IEU UKB)	Associative evidence	Ye et al. (2025)
Heart failure	Activates the NRF2/GPX4 axis to inhibit ferroptosis in cardiomyocytes, mitigating MI/R injury exacerbated by depression	Indole-3-propionic acid (IPA)	*Clostridium sporogenes, Peptostreptococcus* spp.	In vivo + MI/R model with FMT, metagenomics/metabolomics+ in vitro study	Animals: C57BL/6 male mice *n* = 3–10/group (CSDS vs. control, all cardiac/behavioral endpoints); FMT *n* = 3–6/group; metagenomics CON *n* = 7 vs. CSDS *n* = 8; serum targeted tryptophan metabolomics *n* = 4–6/group; IPA supplementation *n* = 3–6/group; TDD survival study *n* = 3–30/group. Cells: H9c2 and HL-1, *n* = 3 independent experiments	Mechanism-suggestive evidence	Mu et al. (2025)
Heart failure	Binds to AhR to upregulate SIRT3 expression, improving diastolic function and metabolic remodeling in an HFpEF model	Indole-3-propionic acid (IPA)	*Clostridium sporogenes, Peptostreptococcus* spp.	Animal study (HFpEF mouse model) + two human population cohorts	Animals: C57BL/6 J male mice *n* = 4–13/group (baseline phenotyping *n* = 8–13; IPA supplementation Chow *n* = 4 / HFpEF *n* = 8; Sirt3-KD and Nnmt-OE *n* = 7/group; Nnmt-ASO control *n* = 9 vs. *n* = 10; therapeutic regimen chow *n* = 8 / HFpEF *n* = 8 / HFpEF+IPA *n* = 12). Cells: HL-1, 5 independent experiments. Human: Cohort 1 *n* = 96 (HFpEF 48 vs. non-HF controls 48, Cleveland Clinic); Cohort 2 *n* = 55 (non-HF 13 / HFpEF 22 / HFrEF 20, Alfred Hospital, Melbourne).	Animal study + associative evidence (human cohorts)	Wang et al. (2024)
Intracranial aneurysms	Plasma IS levels combined with a multivariate model can effectively distinguish ruptured from unruptured aneurysms (AUC = 0.97)	Indoxyl sulfate (IS)	*E. coli, Bacteroides* spp.	two-center cross-sectional, multi-omics+ Animals	Animals: IA incidence & rupture *n* = 11/group plasma IS *n* = 7/6/6 expression *n* = 4/group; Human: Total *n* = 519 (358 UIA + 161 RIA, two regions). Metagenomics *n* = 109 (59 UIA + 50 RIA, Zhujiang); untargeted metabolomics *n* = 60 (30 UIA + 30 RIA, Zhujiang); targeted metabolomics: discovery *n* = 259 (162 UIA + 97 RIA, Zhujiang) + external validation *n* = 244 (195 UIA + 49 RIA, Beijing Tiantan).	Associative (note the risk of overfitting)	Sun et al. (2024)
Aortic dissection	Inhibits VSMC phenotypic switching and ECM degradation, significantly reducing rupture rate in a mouse model (90% → 45%)	Indole-3-carboxaldehyde (3-IAld)	*Lactobacillus* spp. (e.g.*, L. reuteri, L. acidophilus*)	In vivo + in vitro mechanistic study	Animals: Control *n* = 6; 3-IAId *n* = 6; BAPN *n* = 20; BAPN+3-IAId *n* = 20. Mac2/Ly6G infiltration *n* = 9 (figure legend, per-group not stated); EVG elastin quantification *n* = 6 (Control/3-IAId) and *n* = 20 (BAPN groups). Cells: hVSMC, *n* = 5 per group	Mechanism-suggestive evidence	Huang et al. (2023)
Prognosis / biomarkers	Elevated levels of various indole metabolites are independently associated with 3-year MACE and all-cause mortality (after adjustment for traditional risk factors)	Indole, IS, 5-hydroxyindole-3-acetic acid, etc.	*Indole / IS: E. coli, Bacteroides* spp.*; 5-HIAA: host serotonin metabolism* (*gut microbiota-modulated*)	Large-scale prospective cohort (US + European multicentre)	Human: Total *n* = 4,833 (US GeneBank *n* = 4,000; EU LipidCardio *n* = 833); Antibiotic-intervention arm: *n* = 10 healthy volunteers.	Causal (large prospective cohort, multivariable-adjusted)	Nemet et al. (2023)
Prognosis / biomarkers	Reduced gut microbiota capacity to produce AhR ligands may serve as an integrated functional biomarker for metabolic syndrome and cardiovascular risk	Total AhR ligand-producing capacity	*Lactobacillus, Bifidobacterium, Clostridium*	Clinical (cross-sectional; three Paris hospital cohorts) + animal+cells	Animals: HFD vs. CD mice — intestinal transcripts *n* = 5–6/group. Ficz in HFD mice: OGTT *n* = 12–20/group, ITT *n* = 6–10/group. Ficz in ob/ob mice: OGTT *n* = 14–15/group, ITT *n* = 7–8/group. *L. reuteri* supplementation: OGTT *n* = 8/group, ITT *n* = 7–8/group. LPS translocation (Ussing) *n* = 6–12/group; Gcg expression *n* = 6–8/group. Germ-free colonization: 2 groups (CD/GF vs. HFD/GF), n per group not stated. Human: *n* = 127 total (three cohorts pooled; obese BMI > 30 vs. non-obese BMI < 30, also stratified by T2D and hypertension); AhR reporter assay *n* = 127; metabolite dosage missing for 4 subjects (3 obese, 1 non-obese).	Animal study + associative evidence	Natividad et al. (2018)
Dietary intervention	Mixed tree nuts (MTNs) re-program tryptophan metabolic flux: inhibiting the KYN pathway and upregulating indole and 5-HT pathways	Dietary intervention (mixed nuts)	N/A	Human randomized, controlled, two-arm parallel trial	Human: Total *n* = 95 overweight/obese adults — MTNs *n* = 56 vs. pretzels *n* = 39 (fecal analysis *n* = 38 in pretzel group; 1 subject had insufficient plasma). Baseline correlation analysis *n* = 94	Associative evidence	Yang et al. (2023)
Dietary intervention	Flexitarian (plant-protein-based) diets increase circulating protective IPA and reduce the uraemic toxin IS	Dietary intervention (plant-protein diet)	N/A	Human randomized, controlled, crossover feeding trial:	Human: *n* = 19 men participants with complete multi-omics samples *n* = 15; SCFA analysis *n* = 19; β-diversity *n* = 15	Associative evidence	Lépine et al. (2026)
Targeted therapy	Oral supplementation with *Akkermansia muciniphila* or IAA reverses AS plaque progression in Psrc1-deficient mice (AhR-dependent)	Indole-3-acetic acid (IAA)	*Akkermansia muciniphila*	Animal intervention + human validation	Animals mice/cells *n* = 6–10/group (baseline *n* = 6; intervention *n* = 10, atorvastatin control *n* = 6; molecular *n* = 3–6); IAA toxicity experiments not reported; macrophages *n* = 3–6. Cohort *n* = 103 (CAD 77 vs. control 26)	Mechanism-suggestive evidence	Wu et al. (2026)
Traditional Chinese medicine	Paeoniflorin improves atherosclerosis in a single animal model via gut microbiota-Trp metabolism axis	Paeoniflorin	N/A	Single animal model (ApoE^−^/^−^ mice, high-fat diet)	Animals: 48 mice randomized (8 per group)	Mechanism-suggestive evidence	Xia et al. (2025)
Traditional Chinese medicine	Naomaitong ameliorates cerebral and intestinal injury in ischaemic stroke via modulation of the gut-brain axis	Naomaitong / *Akkermansia muciniphila*	*Akkermansia muciniphila*	Single animal model (SD rat MCAO/R model) + untargeted LC–MS metabolomics of CSF/plasma/colon plus targeted 5-HT-pathway quantification; 16S rRNA sequencing; mechanistic validation via AKK supplementation and antibiotic cocktail	Male SD rats. Main experiment: 6 groups × *n* = 15. AKK validation: 30 rats in 4 groups	Mechanism-suggestive (not validated in humans)	Liu et al. (2025)
Traditional Chinese medicine	Aucubin inhibits endothelial-mesenchymal transition (EndMT) via gut microbiota–mediated Trp metabolism (single animal model)	Aucubin / Indole-3-acrylic acid (IAcrA)	N/A	Single animal model (ApoE^−^/^−^ mice)	Animals: Main experiment 5 groups × *n* = 12 (60 mice); FMT 2 groups × *n* = 12 (24 recipients); IAA validation 4 groups × *n* = 12 (48 mice). Figure-level n: serum lipids / NO / ET-1 *n* = 12; aortic qRT-PCR *n* = 5; histology quantification (oil red O, H&E, Masson) *n* = 3; IF staining *n* = 3; Western blot *n* = 4–9 (Au *n* = 4, FMT *n* = 6, IAA *n* = 6; VCAM1/ICAM1 *n* = 9); FMT-group metabolomics & IAA quantification *n* = 8.	Mechanism-suggestive evidence	Luo et al. (2024)

## 3 Core tryptophan metabolic pathways modulated by the gut microbiota

### 3.1 Gut microbiota-dependent pathways of tryptophan metabolism

Tryptophan is an essential amino acid for the human body, and its metabolism is significantly dependent on the gut microbiota. Symbiotic microorganisms convert Trp into various active metabolites by expressing specific enzymes such as tryptophanase (TnaA), thereby forming metabolic pathways independent of the host (Sun et al., 2024; Jia et al., 2024). Current research has established that gut microbiota-mediated Trp metabolism primarily proceeds via three core pathways: the indole pathway, the kynurenine pathway and the serotonin (5-HT) pathway (Hou et al., 2023).

The indole pathway is dominated by Gram-negative bacteria such as *Escherichia coli* and Bacteroides, which catalyze the breakdown of Trp to produce indole and its derivatives (Chen et al., 2024). Among these, the tryptophanase encoded by *E. coli* is the key rate-limiting enzyme in this pathway, catalyzing the conversion of Trp to indole; its expression levels and activity directly determine the flux of indole production in the gut, thereby influencing the levels of downstream host metabolites such as IS (Zhang Y. et al., 2025). Most indole metabolites are natural ligands for the aryl hydrocarbon receptor (AhR); by activating AhR, they can induce interleukin-22 (IL-22), thereby maintaining the intestinal barrier and local immune homeostasis (Yan et al., 2023; Kou et al., 2024; Russo et al., 2024). For example, IPA and indole-3-carboxaldehyde (3-IAld) improve barrier function in colitis models (Kou et al., 2024), while indole-3-lactic acid (ILA) exhibits anti-inflammatory effects in asthma models (Wang H. et al., 2024).

The kynurenine pathway is primarily initiated by indoleamine 2,3-dioxygenase 1 (IDO1) or tryptophan 2,3-dioxygenase (TDO) in host cells, but the gut microbiota can indirectly influence this pathway by regulating IDO1 activity. A high-fat, high-cholesterol diet upregulates intestinal IDO1, leading to increased conversion of Trp to KYN, while simultaneously reducing 5-HT and butyrate, thereby disrupting the intestinal barrier and exacerbating atherosclerosis (Delk et al., 2025). KYN and its downstream products (kynurenic acid, KYNA, and quinolinic acid) are elevated in inflammatory states and are closely associated with obesity, insulin resistance and atherosclerosis (Teunis et al., 2022).

The 5-HT pathway is also regulated by the gut microbiota. A deficiency of intestinal IDO1 can lead to elevated 5-HT levels and exacerbate inflammation (Delk et al., 2025); a high dietary fiber intake promotes the conversion of Trp to 5-HT, thereby enhancing insulin sensitivity (Li Y. et al., 2024). In addition to acting as a neurotransmitter, 5-HT can also locally influence vascular tone and platelet aggregation in the gut, thereby indirectly participating in cardiovascular regulation (Yan et al., 2022). Tryptophan can be further metabolized via the 5-HT pathway to form melatonin. In addition to its role in regulating the circadian rhythm via the pineal gland, melatonin is also synthesized locally in the gut by enterochromaffin cells, and its concentration in the intestinal mucosa can reach 10 to 400 times that found in plasma. Recent studies have revealed a bidirectional interaction between the gut microbiota and host melatonin: typical gut commensal bacteria rarely secrete detectable amounts of melatonin directly; however, their metabolites (short-chain fatty acids, lactic acid and Trp derivatives) can significantly regulate 5-HT and melatonin production in the host’s intestinal chromaffin cells, forming a “microbiota–tryptophan–melatonin” regulatory axis. Clinical cohorts have shown that patients with arrhythmias exhibit gut dysbiosis and bile acid remodeling; this is consistent with the melatonin-sensitive antiarrhythmic effects, suggesting that this pathway plays a potential regulatory role in cardiovascular electrophysiological homeostasis (Tavartkiladze et al., 2026).

Recently, a novel class of tryptophan-derived metabolites—microbial amino-acid-conjugated bile acids (MABAs)—has been identified in human samples. Among these, tryptophan-conjugated cholic acid (Trp-CA) is the most significantly decreased MABA in patients with type 2 diabetes (T2D), and its abundance negatively correlates with clinical glycemic markers (Lin J. et al., 2025). Mechanistically, Trp-CA acts as a ligand for the orphan G protein-coupled receptor MRGPRE, activating both Gs-cAMP and β-arrestin-1-ALDOA signaling pathways to improve glucose tolerance in diabetic mice. Notably, bacterial bile salt hydrolase/transferase from Bifidobacterium is responsible for Trp-CA production (Lin J. et al., 2025), establishing a direct link between a specific gut microbial enzyme and the generation of this bioactive tryptophan conjugate. This discovery expands the known repertoire of gut microbiota-derived tryptophan metabolites and identifies Trp-CA-MRGPRE axis as a potential therapeutic target for metabolic diseases such as T2D, which shares common pathophysiological pathways with cardiovascular disease.

### 3.2 Metabolic flux allocation and its regulatory hubs

Apart from the independent operation of these three pathways, the manner in which a single Trp molecule is diverted to the aforementioned pathways under different conditions—that is, metabolic flux allocation—is a key upstream event determining the ultimate biological effect; however, existing research has paid insufficient attention to this aspect. Metabolic flux allocation is regulated by multiple factors. Firstly, the composition of the microbiota: the abundance of indole-producing bacteria directly determines the flux in the indole pathway. Secondly, host enzyme activity: inflammatory factors such as interferon-γ can induce the expression of IDO1, causing a shift of Trp toward the KYN pathway (Lin T. Y. et al., 2025); furthermore, competition for dietary substrates must not be overlooked; a high-Trp diet can simultaneously saturate multiple pathways, but microorganisms prioritize the utilization of Trp for indole production, and the host’s KYN pathway is only significantly enhanced when microbial metabolic capacity is saturated or impaired. Disruption of this metabolic switch—such as the upregulation of IDO1 caused by a high-fat diet—can simultaneously lead to a reduction in protective indole metabolites and an increase in pro-inflammatory kynurenine products, resulting in a dual-hit effect (Zhou et al., 2024; Sinha et al., 2024; Mondanelli et al., 2021).

## 4 Core mechanisms by which gut microbiota-derived tryptophan metabolites regulate cardiovascular function

Gut microbiota-derived tryptophan metabolites regulate cardiovascular function through a multi-target mechanism. The core pathways include: enhancing the intestinal barrier via the aryl hydrocarbon receptor, reducing endotoxin translocation and systemic inflammation; modulating the Th17/Treg balance to exert bidirectional immune effects, wherein certain metabolites such as indoxyl sulfate can exacerbate vascular injury; and influencing vascular tone and blood pressure through peripheral and central serotonin receptors, with sex-dependent effects. These three mechanisms synergize through the gut-immune-vascular axis, collectively contributing to the development and progression of cardiovascular diseases (Figure 1).

**Figure 1:**
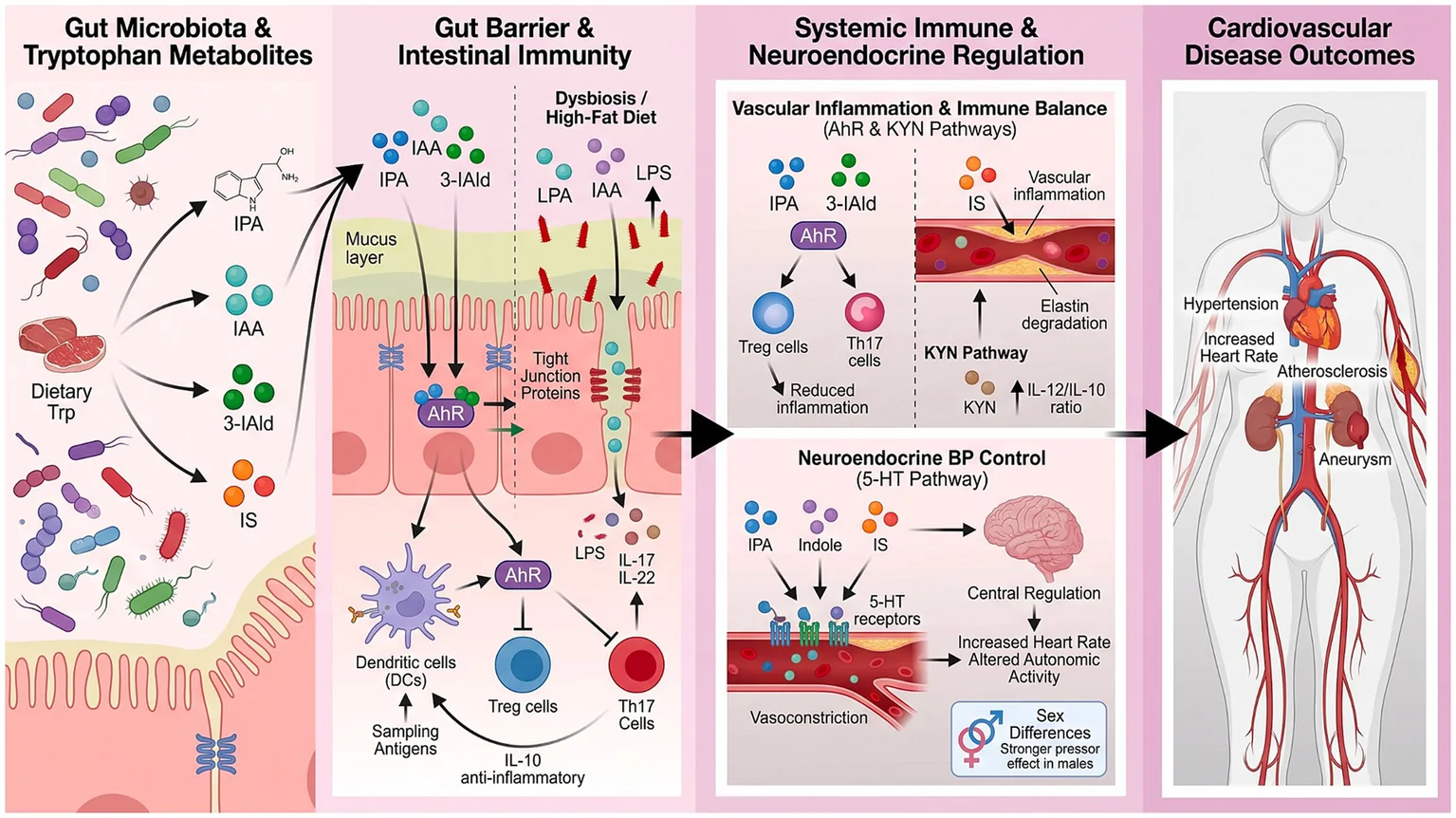
Three core mechanisms by which gut microbiota-derived tryptophan metabolites modulate cardiovascular function. (1) Intestinal barrier maintenance: Microbially-derived indole metabolites (IPA, IAA, 3-IAld) activate the aryl hydrocarbon receptor (AhR) in intestinal epithelial cells, upregulating tight junction proteins (e.g., Occludin, ZO-1) and reducing gut permeability. This limits the translocation of bacterial endotoxins (e.g., LPS) into the circulation, thereby inhibiting systemic inflammation and atherosclerosis progression. A high-fat diet or dysbiosis impairs this protective pathway. (2) Immune and inflammatory regulation: IPA and 3-IAld, as AhR agonists, inhibit pro-inflammatory Th17 cell differentiation while promoting regulatory T cell (Treg) generation, exerting anti-inflammatory effects. Conversely, IS promotes elastin degradation in the vascular wall and releases inflammatory cytokines, exacerbating vascular injury. Under hypertensive conditions, Trp metabolism shifts toward the host-driven KYN pathway, leading to immune imbalance. (3) Neuroendocrine and blood pressure regulation: IPA, indole, and IS regulate vascular tone, heart rate, and autonomic nervous system output through peripheral and central serotonin (5-HT) receptor signaling pathways, exerting bidirectional blood pressure modulation. This effect can be blocked by 5-HT receptor antagonists and exhibits significant sex-dependent differences. The 5-HT pathway itself is host-driven (enterochromaffin cells) but is modulated by gut microbial metabolites and dietary factors.

### 4.1 Regulatory role in maintaining gut barrier integrity

Gut microbiota-derived Trp metabolites play a key role in maintaining intestinal barrier integrity, thereby indirectly influencing cardiovascular function. Several studies have shown that indole derivatives such as IPA, IAA and 3-IAld can enhance intestinal barrier function by activating the AhR signaling pathway, thereby upregulating the expression of tight junction proteins in intestinal epithelial cells (Cui et al., 2026). Under conditions of a high-fat diet or inflammation, dysbiosis of the gut microbiota can lead to reduced levels of Trp metabolites, thereby triggering dysfunction of the intestinal epithelial barrier, manifested as increased intestinal permeability and endotoxemia; the latter has been shown to promote systemic inflammation and the progression of atherosclerosis (Yan et al., 2023; Du et al., 2024). Furthermore, significantly reduced plasma IPA levels have been observed in obese individuals, while these levels rebound following gastric bypass surgery; animal studies have further demonstrated that oral administration of IPA effectively improves high-fat diet-induced intestinal barrier permeability, and *in vitro* experiments have also confirmed that IPA can reverse the increased permeability of T84 intestinal epithelial monolayers induced by pro-inflammatory cytokines (such as interferon-γ) (Jennis et al., 2018). These results suggest that Trp metabolites derived from the gut microbiota play a fundamental role in cardiovascular protection by maintaining the structure and function of the intestinal barrier, thereby reducing the entry of bacterial endotoxins into the bloodstream.

### 4.2 Mechanisms of regulation of immune and inflammatory pathways

Gut microbiota-derived Trp metabolites exert dual anti-inflammatory or pro-inflammatory effects in the cardiovascular system by modulating innate and adaptive immune responses. On the one hand, metabolites such as IPA and 3-IAld act as AhR ligands; upon activation of this receptor, they inhibit the differentiation of pro-inflammatory Th17 cells and promote the generation of regulatory T cells (Tregs), thereby alleviating tissue inflammatory responses (Baranwal et al., 2023). In a mouse model of hypertension induced by N-nitro-L-arginine methyl ester (L-NAME) and a high-salt diet, supplementation with IPA not only reduced systolic blood pressure but also significantly decreased Th17 cell infiltration in the kidneys and increased the proportion of Tregs, suggesting that it improves sodium balance and blood pressure control by modulating the renal immune microenvironment (Baranwal et al., 2023). On the other hand, certain Trp metabolites, such as IS, exhibit pro-inflammatory properties. In patients with ruptured intracranial aneurysms, plasma IS levels are significantly elevated, and animal studies have confirmed that IS exacerbates the degradation of elastin in the aneurysm wall, promoting vascular inflammation and structural damage; antibiotic-induced depletion of the gut microbiota reduces IS levels and decreases aneurysm formation, while exogenous supplementation with IS reverses this protective effect (Sun et al., 2024). Furthermore, in the general population, patients with hypertension have reduced levels of indole-producing bacteria in the gut, and Trp metabolism shifts toward the KYN pathway, leading to elevated serum KYN levels that correlate positively with the IL-12/IL-10 ratio, reflecting a systemic immune imbalance (Shah et al., 2025). In summary, various Trp metabolites play complex and crucial roles in the regulation of cardiovascular inflammation by modulating AhR activity, the Th17/Treg balance and the release of inflammatory cytokines.

Macrophage efferocytosis triggers an IDO1-dependent tryptophan metabolic cascade that promotes inflammation resolution. Upon apoptotic cell clearance, macrophages upregulate tryptophan transport and metabolism, increasing kynurenine production and activating AhR. This IDO1-Kyn-AhR axis drives IL-10 expression and further enhances efferocytic capacity, establishing a positive feedback loop that sustains resolution. This mechanism contributes to atherosclerosis regression following LDL lowering in mouse models (Sukka et al., 2024), extending the known functions of the tryptophan-AhR axis from T cell regulation to macrophage metabolic reprogramming, and highlighting IDO1 and AhR as potential therapeutic targets for non-resolving chronic inflammatory diseases such as atherosclerosis.

### 4.3 Mechanisms linking neuroendocrine function to blood pressure regulation

Gut microbiota-derived Trp metabolites may also participate in blood pressure regulation via neuroendocrine pathways. Studies have found that intravenous injection of IPA can increase mean arterial blood pressure (MABP) in a dose-dependent manner in both normal and hypertensive rats, accompanied by an increase in heart rate in hypertensive rats; *in vitro* experiments have shown that IPA enhances the contractile response of de-endothelialised mesenteric resistance arteries and inhibits acetylcholine-mediated vasodilation, suggesting that it has a direct pressor effect (Konopelski et al., 2021). Similarly, in anesthetised male Wistar rats, intravenous administration of indole or its sulphated metabolite IS increased both blood pressure and heart rate, whereas intraventricular injection of indole produced a hypotensive effect, indicating that its action involves dual peripheral and central regulatory mechanisms. It is worth noting that the aforementioned cardiovascular effects can be inhibited by the 5-HT receptor antagonists ondansetron and pizotifen, but are not affected by dopamine or α-adrenergic receptor antagonists, suggesting that Trp metabolites primarily mediate blood pressure regulation via the 5-HT signaling pathway (Huć et al., 2018). Furthermore, in a salt-sensitive hypertension model, significant sex differences were observed: oral administration of indole caused an increase in blood pressure only in male rats, while females exhibited a stronger metabolic buffering capacity, suggesting that the Trp–indole pathway involves sex-specific mechanisms in blood pressure regulation (Bardhan et al., 2024). Taken together, this evidence suggests that Trp metabolites derived from the gut microbiota participate in neuroendocrine blood pressure regulation by activating peripheral and central 5-HT receptors and influencing vascular tone and autonomic activity, thereby playing a significant role in the etiology and progression of cardiovascular diseases such as hypertension.

## 5 Research progress on key metabolites in various common cardiovascular diseases

In recent years, the regulatory role of tryptophan metabolites derived from gut microbiota in cardiovascular diseases has become a research hotspot. Substantial evidence indicates that key metabolites such as IS, IAA, IPA, 3-IAld, and the KYN/Trp exhibit complex and diverse pathophysiological effects in diseases including atherosclerosis, hypertension, heart failure, aortic dissection, and coronary microvascular dysfunction by influencing multiple signaling pathways, such as oxidative stress, immune cell polarization, cholesterol reverse transport, vascular smooth muscle phenotype switching, ferroptosis, and mitochondrial function—with some metabolites exerting clear pathogenic roles, while others demonstrate significant protective effects. To systematically organize the core roles and molecular networks of these metabolites in different cardiovascular diseases, this section integrates recent research progress and summarizes them in the Figure 2, providing readers with an intuitive understanding of the regulatory mechanisms discussed in subsequent disease sub-sections.

**Figure 2:**
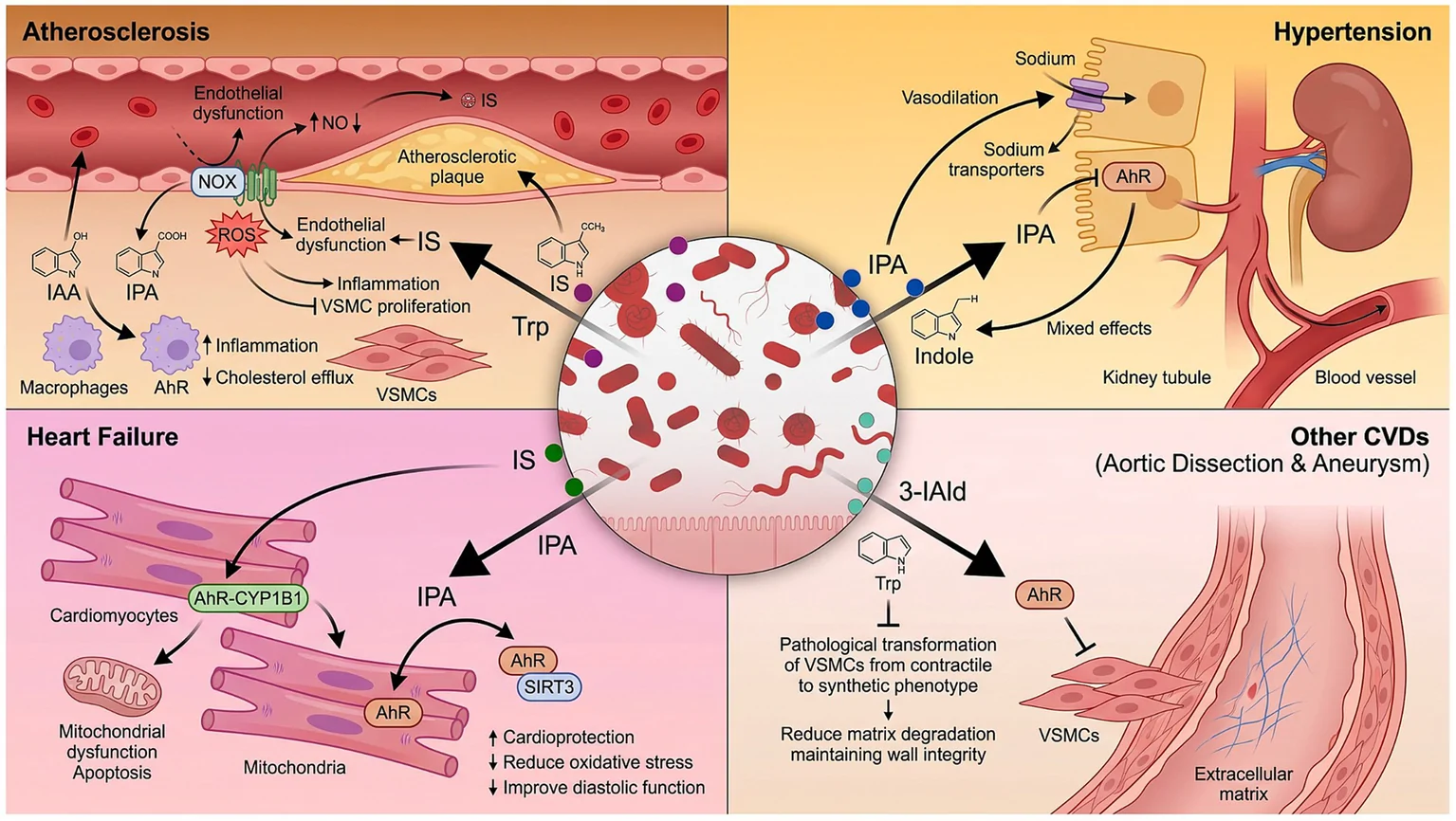
Key regulatory roles and mechanisms of microbiota-derived tryptophan metabolites in cardiovascular diseases. This figure summarizes the main effects and representative molecular pathways of tryptophan metabolites derived from the gut microbiota (IS, IAA, IPA, 3-IAld, and KYN/Trp ratio) in atherosclerosis, hypertension, heart failure, aortic dissection, and coronary microvascular dysfunction.

### 5.1 Atherosclerosis

IS, as a key Trp metabolite derived from the gut microbiota, plays a clear pathogenic role in cardiovascular atherosclerosis. Extensive evidence indicates that IS is a vasotoxic substance that is directly involved in the pathological processes of atherosclerosis, arteriosclerosis, vascular calcification and impaired vascular repair (Paeslack et al., 2022; Konopelski et al., 2021; Wu et al., 2017); clinical studies have shown that serum IS levels are significantly positively correlated with aortic stiffness in patients with coronary artery disease (CAD) and are closely associated with overall mortality and cardiovascular mortality (Lin T. J. et al., 2020). At the mechanistic level, IS induces vascular oxidative stress by activating NADPH oxidase-mediated redox signaling pathways, thereby impairing the nitric oxide (NO)/cGMP signaling pathway and reducing superoxide dismutase (SOD) activity, leading to endothelial dysfunction and impaired vasodilation (Matsumoto et al., 2021); simultaneously, IS acts synergistically in macrophages via the OATP2B1 and Dll4-Notch signaling pathways to exacerbate the vascular inflammatory response (Nakano et al., 2019); furthermore, IS promotes the expression of tumor necrosis factor and cell proliferation in vascular smooth muscle cells (VSMCs) (Ng et al., 2016) and facilitates vascular calcification through METTL14-dependent m6A methylation regulation (Chen et al., 2019), with calcification and matrix degradation being key factors in plaque instability; in uraemic models, IS can also induce arterial thrombosis and inhibit angiogenesis (Yang et al., 2017; Berg et al., 2022; Lin T. Y. et al., 2020), further exacerbating atherosclerotic complications. Conversely, the KYN pathway protects against vascular calcification. IDO1-derived kynurenine inhibits osteogenic reprogramming of VSMCs by promoting RUNX2 degradation via the ubiquitin-proteasome pathway. Mechanistically, kynurenine acts as an endogenous AhR ligand, facilitating cullin 4B-AhR interaction to form an E3 ubiquitin ligase complex that ubiquitinates RUNX2. Clinical evidence shows reduced IDO1 activity and kynurenine levels in patients with coronary artery calcification (Ouyang et al., 2022). This tryptophan-KYN-AhR-RUNX2 axis suggests that kynurenine supplementation or IDO1 activation may offer a therapeutic strategy for preventing arterial calcification. Furthermore, the KYN pathway of Trp is closely associated with the progression of atherosclerosis, and metabolic shifts in this pathway are often accompanied by a systemic inflammatory state (Teunis et al., 2022) (Figure 2). These metabolites predominantly regulate the function of immune cells via the AhR signaling pathway, thereby maintaining intestinal mucosal homeostasis and influencing vascular inflammation.

In recent years, the regulatory role of indole-derived Trp metabolites from the gut microbiota in tumor immunotherapy has also attracted attention, particularly regarding their potential impact on the efficacy of immune checkpoint inhibitors (ICIs) and cardiovascular complications. Although ICI therapy can activate anti-tumor immunity, it may accelerate the progression of atherosclerotic cardiovascular disease; this side effect is closely associated with systemic inflammatory dysregulation. Indole derivatives, as natural ligands of AhR, are known to modulate the Th17/Treg balance and influence dendritic cell maturation and cytokine release, thereby exerting bidirectional regulatory effects in the tumor microenvironment and vascular inflammation (Dormans et al., 2025). Therefore, an in-depth analysis of the interactions within the microbiota–metabolite–immune–vascular axis holds promise for the development of metabolite-based, personalized adjunctive intervention strategies for patients undergoing ICI therapy, with the aim of reducing the risk of cardiovascular events without compromising antitumour efficacy. Although this interdisciplinary field is still in its infancy, it offers a new perspective on the mechanistic study and clinical management of atherosclerosis.

The interaction between host genetic variants and Trp metabolism in the gut microbiota is attracting increasing attention in the context of atherosclerosis. Genome-wide association studies have linked the PSRC1 gene to CAD, while animal experiments indicate that Psrc1 deficiency accelerates atherosclerosis by inducing gut dysbiosis—particularly depletion of *Akkermansia muciniphila*. Mechanistically, Psrc1 deficiency leads to reduced colonic mucin and altered expression of Trp-metabolizing enzymes, resulting in decreased IAA production and inhibition of AhR signaling in macrophages; simultaneously, Psrc1 stabilizes the AhR protein via Uchl3-mediated deubiquitination, a process further compromised by *A. muciniphila*-dependent IAA depletion. Clinical data indicate that PSRC1 downregulation in CAD patients is positively correlated with reduced plasma IAA levels. In intervention studies, oral supplementation with *A. muciniphila* or IAA significantly reversed atherosclerotic lesions in Psrc1-knockout mice; this effect was attributed to the inhibition of macrophage apoptosis within plaques and could be abolished by AhR antagonists (Wu et al., 2026).

With regard to the regulatory mechanisms of the Trp metabolite IAA, the role of the gut commensal *Bacteroides ovatus* has also been elucidated. Through integrated microbiome–metabolome analysis of fecal and serum samples from patients with atherosclerotic cardiovascular disease and individuals with risk factors for coronary heart disease, researchers identified *B. ovatus* as a key functional strain. Fecal microbiota transplantation experiments confirmed that this strain alone can produce anti-atherosclerotic effects comparable to those of donor feces. At the mechanistic level, *B. ovatus* exerts its effects primarily by restoring the intestinal barrier and enhancing bile acid metabolism; the IAA it produces inhibits the TLR4/MyD88/NF-κB pathway in M1 macrophages, promotes M2 macrophage polarization, restores the M1/M2 balance, and ultimately alleviates aortic inflammation (Liu W. et al., 2025). These findings establish *B. ovatus* for the first time as a second-generation probiotic candidate strain that exerts anti-atherosclerotic effects via IAA, providing a new tool for precision intervention strategies targeting Trp metabolism.

With regard to atherosclerotic thrombosis, the pathogenic role of the gut microbiota is also supported by multi-omics evidence. Metagenomic and serum metabolomic analyses of a cohort of CAD patients revealed significant alterations in both gut microbiota composition and serum metabolic profiles during CAD progression, with the genus Bacteroides contributing to disease development via the lipopolysaccharide, menadione and methane production pathways. Of particular note is that coronary artery thrombosis is characterized by elevated circulating L-Trp levels; this change is associated with the abundance of *Bacteroides thetaiotaomicron*, a species with a high potential for Trp biosynthesis. Transplantation experiments in germ-free mice confirmed that colonization by this single bacterium alone can induce thrombosis, exacerbate platelet hyperreactivity and elevate fecal L-Trp levels, suggesting that an increased Trp load of microbial origin may directly contribute to the occurrence of thrombotic events by affecting platelet function (Liu et al., 2024). These findings extend the scope of Trp research from “microbial metabolites” to the “amino acid precursor” itself, revealing a novel mechanistic pathway by which the gut microbiota influences thrombosis through the regulation of the L-Trp system, and providing a theoretical basis for the development of microbiota-associated biomarkers and targeted interventions for thrombotic events.

In the anti-atherosclerotic mechanism of IPA, in addition to its known anti-inflammatory and antioxidant effects, its role in promoting cholesterol efflux from macrophages has also been revealed. Based on integrated microbiome–metabolome analyses of CAD patients and healthy controls, IPA was identified as the most significantly downregulated microbial Trp metabolite in CAD and validated in an independent cohort (*n* = 256), with its levels showing a negative correlation with atherosclerotic cardiovascular disease (ASCVD) risk and disease severity. Animal studies demonstrated that dietary supplementation with IPA reduces atherosclerotic plaque in ApoE^−^/^−^ mice. At the mechanistic level, IPA promotes cholesterol efflux from macrophages to apolipoprotein ApoA-I by downregulating miR-142-5p and thereby lifting its inhibition of ABCA1, consequently enhancing reverse cholesterol transport; this pathway is dysregulated in macrophages from CAD patients and correlates with changes in circulating IPA levels (Xue et al., 2022) (Figure 2).

At the level of clinical outcomes in atherosclerosis, differences in gut-derived Trp metabolites are also closely associated with disease burden and postoperative risk. Compared with the control group, patients with advanced atherosclerosis (those who had undergone revascularisation or amputation) had significantly lower plasma concentrations of indole, Trp, IPA and 3-IAld, which were positively correlated with the ankle-brachial index (ABI), suggesting that these markers may reflect the overall burden of atherosclerosis; conversely, the KYN/Trp ratio was significantly elevated. Prognostic analysis showed that the baseline KYN/Trp ratio was independently associated with cardiac complications at 30 days post-surgery and long-term major adverse cardiac events (MACE; odds ratio [OR] = 44.1), and this association remained significant even after adjusting for factors such as diabetes. These findings extend the evidence for the protective effects of Trp metabolites from early-stage populations to patients with advanced disease, and reveal for the first time that the KYN/Trp ratio can serve as an independent predictor of postoperative cardiovascular complications (Cason et al., 2018).

### 5.2 Hypertension

The role of Trp metabolites in hypertension is supported by complex and even contradictory evidence. On the one hand, in a mouse model of hypertension induced by L-NAME and a high-salt diet, IPA levels in serum and feces were significantly reduced; a three-week dietary supplementation with IPA effectively reduced systolic blood pressure, increased sodium excretion, and reduced Th17 cell infiltration in the kidneys, while showing a trend toward an increase in Treg cells; *in vitro* experiments confirmed that IPA inhibits Th17 differentiation and promotes Treg generation, suggesting that it improves sodium balance and blood pressure control by regulating renal immune homeostasis (Baranwal et al., 2023).

Women with salt-sensitive hypertension exhibit a greater capacity of the gut microbiota to buffer the Trp–indole pathway, while men are more prone to elevated blood pressure due to increased indole metabolites, suggesting that sex differences may influence the effects of these metabolites (Wu et al., 2017). It is important to note that the experimental context significantly influences IPA’s vascular effects. A study by Konopelski et al. (2021) reported that intravenous administration of IPA at suprapharmacological doses increased MABP in both SHR and WKY rats in a dose-dependent manner, accompanied by tachycardia, and enhanced the contractile response of de-endothelialised mesenteric resistance arteries *in vitro*. These findings appear to contradict the blood pressure-lowering effects observed in other models (Baranwal et al., 2023). However, this discrepancy is likely attributable to the non-physiological administration route (intravenous bolus) and the high doses used, which do not reflect endogenous IPA concentrations achieved through gut-derived production or oral supplementation. Thus, the pressor response to IPA appears to be a pharmacological artifact rather than a physiologically relevant effect. Intravenous administration of indole-3-propionic acid elevates blood pressure in rats through cardiac and vascular mechanisms, including enhanced myocardial contractility and cardiomyocyte metabolic activity, with the pressor response being partially dependent on sympathetic neural pathways (Konopelski et al., 2021). These seemingly contradictory results suggest that IPA may exert different effects under varying pathological conditions, routes of administration or dosages; further clarification is required regarding functional differences at physiological and pharmacological concentrations.

In obese adolescents, an association between gut-derived Trp metabolites and cardiovascular metabolic risk markers has also been preliminarily confirmed. Studies have found that IPA is negatively correlated with diastolic blood pressure, total cholesterol and low-density lipoprotein cholesterol (LDL-C), and that this association is independent of gender and degree of obesity; IS, on the other hand, is positively correlated with fasting blood glucose levels, with adolescents with impaired fasting glucose exhibiting approximately 1.6-fold higher IS levels. Furthermore, indole-3-acetaldehyde levels decline with age, while IAA and indole levels are higher in Hispanic populations than in non-Hispanic populations. The aforementioned metabolites showed no significant association with vascular endothelial function (flow-mediated dilation), cardiopulmonary fitness (peak oxygen uptake) or platelet mitochondrial function (Rivera et al., 2025). These findings suggest that Trp metabolites, particularly IPA and IS, are associated with cardiovascular metabolic risk markers as early as the adolescent stage of obesity, supporting their potential value in the early risk assessment of CVD.

### 5.3 Heart failure

Although direct evidence is relatively limited, existing studies have preliminarily revealed the potential regulatory role of Trp metabolites in heart failure. An *in vitro* study found that IPA can significantly alleviate doxorubicin-induced oxidative stress, inflammatory responses and apoptosis in human cardiomyocytes by inhibiting the HDAC6/NOX2 signaling pathway, suggesting its protective potential in drug-induced myocardial injury and heart failure (Li C. et al., 2024). Furthermore, heart failure is often accompanied by impaired intestinal barrier function, leading to the translocation of bacteria and their metabolites into the bloodstream, thereby exacerbating a systemic inflammatory state; however, indole compounds derived from the gut microbiota can maintain intestinal barrier integrity by activating AhR, thereby indirectly alleviating heart failure-associated inflammation (Yang et al., 2026). Concurrently, an overall imbalance in Trp metabolism is also observed in heart failure-related pathological states (Mohiti et al., 2025); for instance, IS levels in patients with end-stage renal disease are significantly associated with specific energy metabolism pathways (Sato et al., 2016), while the accumulation of uraemic toxins (including indole derivatives) in patients with chronic kidney disease (CKD) can activate AhR, promoting oxidative stress and vascular calcification, thereby indirectly affecting cardiac function (Liu et al., 2021; Dou et al., 2018). In heart failure associated with CKD, IS has been identified as a key effector molecule driving the deterioration of cardiac function. The mechanism involves *E. coli* converting Trp into indole via tryptophanase (TnaA); following its metabolism into IS by the liver, this activates the AhR-CYP1B1 axis in cardiomyocytes, specifically impairing mitochondrial function and inducing myocardial apoptosis (Lu et al., 2024). Mendelian randomization studies further support a causal association between the gut microbiota and its derived metabolites and heart failure, with certain circulating Trp metabolites potentially acting as mediators in the disease process (Ye et al., 2025).

The regulatory role of Trp metabolites in depression-exacerbated myocardial ischaemia/reperfusion (MI/R) injury has also been elucidated. Studies have found that fecal microbiota transplantation in depressed mice exacerbates cardiac dysfunction and adverse ventricular remodeling following MI/R, suggesting that dysbiosis is a key mediator of depression-exacerbated myocardial injury. Metabolomic analysis revealed that alterations in the structure and composition of the gut microbiota during depression lead to dysregulation of Trp metabolism, with a significant reduction in IPA levels. Mechanistically, IPA exerts a protective effect against MI/R injury by inhibiting ferroptosis in cardiomyocytes via activation of the NRF2/System xc^−^/GPX4 signaling axis (Mu et al., 2025). Furthermore, IPA protects against MI/R injury by inhibiting cardiomyocyte ferroptosis via the NRF2/System xc^−^/GPX4 axis. Depression-induced gut dysbiosis reduces IPA levels, thereby exacerbating MI/R injury and impairing long-term cardiac function (Mu et al., 2025). This ‘brain-gut-heart axis’ mechanism suggests that IPA supplementation or microbiota modulation may improve post-MI/R outcomes in depressed patients. IPA also exerts anti-fibrotic effects (Mu et al., 2025), indicating broader potential in cardiovascular remodeling.

The protective effect of IPA has also been validated in heart failure with preserved ejection fraction (HFpEF). IPA levels were significantly reduced in the plasma and cardiac tissue of mice in an HFpEF model; dietary supplementation with IPA alleviated diastolic dysfunction, metabolic remodeling, oxidative stress, systemic inflammation and intestinal barrier damage. At the mechanistic level, IPA upregulates SIRT3 expression in cardiac muscle by binding to AhR, thereby restoring nicotinamide, NAD^+^/NADH levels and the activity of the NAD^+^ salvage pathway; SIRT3 knockout abolishes the protective effect of IPA on diastolic function, confirming this pathway as its core mechanism of action. In two patient cohorts, circulating IPA levels were significantly reduced in patients with HFpEF, consistent with findings in animal models, supporting its protective function in humans (Wang Y. C. et al., 2024).

### 5.4 Other common cardiovascular diseases

In addition to the three major disease categories mentioned above, Trp metabolites derived from the gut microbiota also play a role in other cardiovascular-related diseases. For example, in patients with both unruptured and ruptured intracranial aneurysms, plasma IS levels are significantly elevated and positively correlated with the abundance of indole-producing bacteria and the expression of tryptophanase; animal models have confirmed that IS contributes to aneurysm rupture by promoting the degradation of the vascular wall (Sun et al., 2024). Furthermore, research into diabetic cardiomyopathy suggests that gut microbiota metabolites may influence myocardial structure and function by modulating oxidative stress, inflammation and apoptosis, with Trp metabolism potentially constituting one such mechanism (Yuan et al., 2022). With regard to stroke, observational studies have found that alterations in the composition of patients’ gut microbiota are associated with larger infarct volumes and poorer clinical outcomes; although Trp metabolites were not directly measured, considering the overall role of the microbiota–metabolome axis, it is hypothesized that they may be involved in the process of neurovascular damage (Bardhan et al., 2024). Although some studies have focused on pulmonary fibrosis, osteoporosis or infectious diseases (such as pneumonia), finding that Trp metabolites such as IAA or IPA exert effects in these conditions via the AhR or mTOR pathways (Kullberg et al., 2025; Li J. et al., 2024; Huang et al., 2023), their broader implications for the cardiovascular system must still be interpreted with caution, as direct experimental evidence specifically addressing other cardiovascular diseases is currently lacking. Overall, current data support the notion that Trp metabolites exert broad yet heterogeneous regulatory effects across various cardiovascular pathological states; this requires validation through disease-specific mechanistic studies.

Coronary microvascular dysfunction (CMD) is a key pathological basis of ischaemic heart disease, and links to the gut microbiota–metabolome axis are beginning to emerge. Animal studies indicate that under CMD conditions, the structure and diversity of the gut microbiota are significantly altered, and the expression levels of Trp metabolic pathways are markedly downregulated. Zacopride, which exerts dual regulatory effects on 5-HT₄ receptors and Kir2.1 channels, partially restores coronary flow reserve in CMD rats and upregulates the chlorophyll a metabolic pathway; however, it does not significantly restore the Trp metabolic pathway, suggesting that the pathogenesis of CMD may involve a regulatory imbalance in the “chlorophyll/haem–tryptophan metabolic axis”. Furthermore, genera and species such as JC017, Chromelosporium and Barnesiella may serve as characteristic microbial biomarkers of CMD (Chen et al., 2025). The above analysis reveals that zacopride exerts its therapeutic effects by reshaping microbiota–host interactions and regulating this metabolic axis, providing a new perspective for research into the microbial metabolic mechanisms of CMD.

In aortic dissection (AD)—a vascular emergency characterized by acute onset and extremely high mortality—breakthrough progress has also been made in evidence for direct intervention with Trp metabolites derived from the gut microbiota. In the BAPN-induced mouse aortic dissection model, the gut-derived tryptophan metabolite 3-IAId reduced the overall incidence of dissection (including rupture) from 90 to 45%, and the rupture-related mortality from 55 to 20%; its mechanism is associated with inhibiting the phenotypic switch of vascular smooth muscle cells from a contractile to a synthetic phenotype, attenuating extracellular matrix degradation, and reducing macrophage infiltration and inflammatory cytokine expression (Huang et al., 2023). At the mechanistic level, this protective effect is closely related to the aforementioned regulation of VSMC phenotypes and the maintenance of extracellular matrix (ECM) homeostasis: 3-IAld effectively inhibits the pathological transformation of VSMCs from a contractile to a synthetic phenotype, reduces the rupture of elastic fibers and collagen degradation within the aortic wall, and simultaneously suppresses macrophage recruitment and the release of inflammatory factors, thereby delaying the onset and progression of AD in multiple ways (Huang et al., 2023).

These findings are further corroborated by an independent study demonstrating that indole-3-aldehyde (3-IAId), a tryptophan metabolite with structural similarity to 3-IAld, exerts therapeutic effects in a BAPN-induced TAD mouse model (Yu et al., 2025). Mechanistically, 3-IAId similarly attenuates aortic dissection progression by inhibiting VSMC phenotypic switching from a contractile to a synthetic phenotype, reducing extracellular matrix degradation, and suppressing macrophage infiltration and inflammatory cytokine expression (Yu et al., 2025). The convergent evidence from these independent studies strengthens the notion that indole-containing tryptophan metabolites represent a promising class of protective molecules against aortic dissection, acting through conserved pathways that regulate VSMC homeostasis and vascular wall integrity.

## 6 Exploration of novel biomarkers in the diagnosis of cardiovascular diseases

### 6.1 The potential value of tryptophan metabolites as biomarkers

In recent years, Trp metabolites derived from the gut microbiota have gradually been recognized as novel biomarkers with potential for clinical translation, owing to their multifaceted regulatory roles in the etiology and progression of cardiovascular disease. These metabolites not only contribute to maintaining gut barrier integrity and regulating systemic immune homeostasis, but also directly influence key pathophysiological processes such as vascular endothelial function, inflammatory responses and blood pressure regulation (Olić et al., 2026; Kondapalli et al., 2025). As previously mentioned, indole derivatives such as IPA, IAA and IS have been shown to modulate host immunity and inflammatory states via the AhR signaling pathway, exerting dual pro-inflammatory or anti-inflammatory effects in various cardiovascular diseases, including atherosclerosis, hypertension and heart failure (Paeslack et al., 2022; Liu et al., 2021). Activation of the KYN pathway reduces the availability of Trp for the production of beneficial microbiota-derived indole compounds and 5-HT, thereby leading to impaired intestinal barrier function, exacerbated systemic inflammation and accelerated atherosclerotic progression, suggesting that metabolites associated with this pathway also have potential as biomarkers.

Furthermore, studies on gut microbiota–host interactions have shown that changes in the types and concentrations of Trp metabolites can reflect the structural and functional state of the gut microbiota, thereby enabling an indirect assessment of an individual’s cardiovascular health (Nesci et al., 2023). Consequently, considering their biological functions, metabolic stability and strong correlation with disease progression, Trp metabolites derived from the gut microbiota possess both the theoretical basis and practical potential to serve as novel, non-invasive, dynamically monitorable cardiovascular biomarkers.

### 6.2 Analysis of existing clinical evidence and diagnostic performance

#### 6.2.1 Preliminary evidence from cross-sectional and case–control studies

Although most mechanistic evidence originates from animal models, several clinical studies have preliminarily validated the practical efficacy of Trp metabolites in the diagnosis of cardiovascular disease. A cross-sectional study based on the Qatar Biobank database compared the metabolic profiles of 112 patients with confirmed CVD with those of 112 matched controls. It found that the indole-acetyl-glutamine pathway was significantly enriched in the patient group, while levels of the cardioprotective metabolite IPA were reduced, and levels of the pro-inflammatory metabolite IAA and indole-acetyl-glutamine were elevated, suggesting that this metabolic axis may serve as a potential marker for disease risk assessment (Naja et al., 2025). Another study integrating metabolomics with fecal metagenomics focused on patients with intracranial aneurysms. The results showed that plasma IS levels were significantly higher in patients with ruptured aneurysms (RIA) than in those with unruptured aneurysms (UIA), and that tryptophanase expression was up-regulated and the abundance of indole-producing bacteria was increased in the gut microbiota; a multivariate logistic regression model constructed using IS and other microbe-derived Trp metabolites (after adjusting for age, sex, hypertension, diabetes and smoking history) achieved an AUC of 0.97 for distinguishing RIA from UIA in an independent external cohort, demonstrating excellent diagnostic discriminatory ability. It should be noted that, while this AUC was derived from a multivariate model and validated in an independent cohort, the sample sizes were relatively limited (*n* = 98 in the discovery cohort and *n* = 60 in the validation cohort). The excellent discriminatory performance may carry a certain risk of overfitting, and its generalisability to larger, multicentre populations remains to be further verified (Sun et al., 2024).

#### 6.2.2 Causal evidence from prospective cohort studies

In prospective cohort studies, phenylacetylglutamine (PAG)—a gut microbiota-derived metabolite closely associated with Trp metabolism—has been shown to be independently associated with future risk of coronary heart disease (CHD). In the Malmö Diet and Cancer – Cardiovascular Cohort (*N* = 3,361) and the Malmö Preventive Project (*N* = 880), after adjusting for traditional cardiovascular risk factors, each unit increase in PAG was associated with a hazard ratio for CHD of 1.17 (95% CI: 1.06–1.29, *p* = 1.9 × 10^−3^) (Ottosson et al., 2020). Furthermore, quantitative analysis of plasma from CVD patients using a derivatisation-based LC–MS/MS method revealed that multiple classes of gut microbiota metabolites, including six Trp metabolites, could be detected simultaneously; levels of some of these metabolites were significantly altered in patients, demonstrating the technical feasibility of establishing a clinical testing platform (Liao et al., 2021).

In the general community population, prospective associations between various Trp metabolites and cardiovascular metabolic risk markers have also been validated. A longitudinal cohort study involving 318 community-dwelling adults found that a 1 μmol/L increase in IAA was associated with a 5.08% reduction in LDL-C; IPA was negatively correlated with brachial-ankle pulse wave velocity (baPWV) (a reduction of 1.32%), suggesting an association with reduced arterial stiffness; 3-IAld was associated with a 1.91% reduction in total cholesterol and a 0.58% increase in the ABI, suggesting a potential protective effect against peripheral arterial disease; however, it was also associated with a borderline reduction in high-density lipoprotein cholesterol (HDL-C) (1.79%). Furthermore, the researchers identified 18 bacterial genera positively correlated with serum IPA concentrations and developed an “IPA predictive microbial score”, which was negatively correlated with baPWV (a reduction of 0.48%) (Pan et al., 2024).

Furthermore, a study based on two independent prospective cohorts from the United States (*n* = 4,000) and Europe (*n* = 833) used stable isotope dilution mass spectrometry to quantitatively analyze aromatic amino acids and their metabolites, systematically evaluating the relationship between gut microbiota-derived metabolites and cardiovascular outcomes. The results showed that various Trp-derived metabolites—including indole (and its downstream metabolites indole-glucuronide and IS), indole-3-pyruvate (converted to indole-3-lactate and indole-3-acetylglutamine) and 5-hydroxyindole-3-acetic acid—were significantly positively correlated with the risk of MACE (defined as myocardial infarction, stroke or death) and all-cause mortality, and these associations remained significant after adjustment for traditional cardiovascular risk factors (including age, sex, body mass index, smoking, hypertension, diabetes, LDL-C and high-sensitivity C-reactive protein [hs-CRP]) (*p* < 0.05 after multiple adjustments), indicating that this class of metabolites provides independent prognostic information beyond existing clinical indicators. Antibiotic intervention studies (in humans and mice) have confirmed that levels of these metabolites decrease significantly following suppression of the gut microbiota, thereby confirming their gut microbiota dependence (Nemet et al., 2023). This study provides high-level epidemiological evidence from a large-scale, multicentre population study that Trp metabolites serve as independent predictors of cardiovascular events, and suggests that combining quantitative metabolomics with clinical risk assessment holds promise for identifying high-risk, individualized targets for intervention.

However, there is currently a lack of large-scale, multicentre, standardized clinical validation studies. Although certain metabolites, such as IAA, have demonstrated prognostic value in non-cardiovascular diseases such as pneumonia (e.g., in patients with severe pneumonia, each log₂ increase in IAA was associated with a hazard ratio of 1.30 for mortality, 95% CI: 1.02–1.68, *p* = 0.037) (Li J. et al., 2024), their specificity in cardiovascular disease requires further clarification.

#### 6.2.3 Novel functional biomarkers: the gut microbiota’s ability to produce AhR ligands

The gut microbiota’s overall capacity to metabolize Trp into AhR ligands has been proposed as a novel functional biomarker. Preclinical and clinical studies have shown that, in the context of metabolic syndrome, the gut microbiota’s ability to produce AhR agonists is significantly reduced; furthermore, this alteration is not a passive consequence of the disease but rather a functionally deficient state that plays a pathologically driving role (Natividad et al., 2018). Unlike measurements of individual metabolite concentrations, this indicator integrates comprehensive information on gut microbiota composition, metabolic enzyme activity and microenvironmental factors, and may provide a more comprehensive reflection of the functional state of the Trp–microbiota–host interaction. Although this concept is currently applied primarily in the fields of metabolic syndrome and hepatic steatosis, its core principle of assessing the “functional output” of the microbiota holds promise for extension to risk stratification in cardiovascular disease, particularly by providing new insights for monitoring the efficacy of personalized microbiota interventions.

#### 6.2.4 Incremental predictive value of tryptophan metabolites compared with traditional biomarkers

Unlike N-terminal pro-B-type natriuretic peptide (NT-proBNP), which primarily reflects myocardial wall tension and the severity of heart failure, Trp metabolites are more closely associated with vascular inflammation, endothelial function and atherosclerotic burden (for example, the KYN/Trp ratio is independently associated with postoperative cardiovascular complications, OR = 44.1 (Cason et al., 2018)); the predictive dimensions of the two are complementary. Compared with LDL-C, IPA influences atherosclerosis via dual pathways—promoting cholesterol efflux (Xue et al., 2022) and exerting anti-inflammatory effects (Baranwal et al., 2023)—and its predictive value may be partially independent of traditional lipid markers. Furthermore, gut microbiota functional biomarkers (such as AhR ligand-generating capacity (Zhang et al., 2017) and the IPA predictive gut microbiota score (Nemet et al., 2023)) integrate multidimensional gut microbiota information and may offer comprehensive risk assessment capabilities that go beyond those of individual metabolites or traditional biomarkers. Nevertheless, there are currently limited reports comparing the direct incremental AUC of Trp metabolites with that of traditional markers; head-to-head comparative studies in a unified cohort are urgently needed to clarify their clinical application.

Overall, while existing clinical evidence tentatively supports the potential of Trp metabolites as biomarkers for cardiovascular disease, their sensitivity, specificity, population generalisability and diagnostic value when combined with traditional markers still require systematic evaluation.

## 7 Treatment strategies for cardiovascular disease based on tryptophan metabolic pathways in the gut microbiota

Various active metabolites produced by the gut microbiota through the Trp metabolic pathway play a bidirectional regulatory role in the occurrence and development of CVD. Some metabolites have clear pathogenic effects, while others exhibit significant vascular protective effects. This finding provides new intervention targets and strategic directions for the prevention and treatment of CVD. In recent years, significant progress has been made in intervention studies targeting the microbiota-Trp metabolic axis, forming a multi-layered therapeutic strategy system encompassing dietary intervention, emerging therapies, active ingredients of traditional Chinese medicine, and re-evaluation of existing drugs. The following is a review of the research progress on the four major categories of intervention strategies (Figure 3).

**Figure 3:**
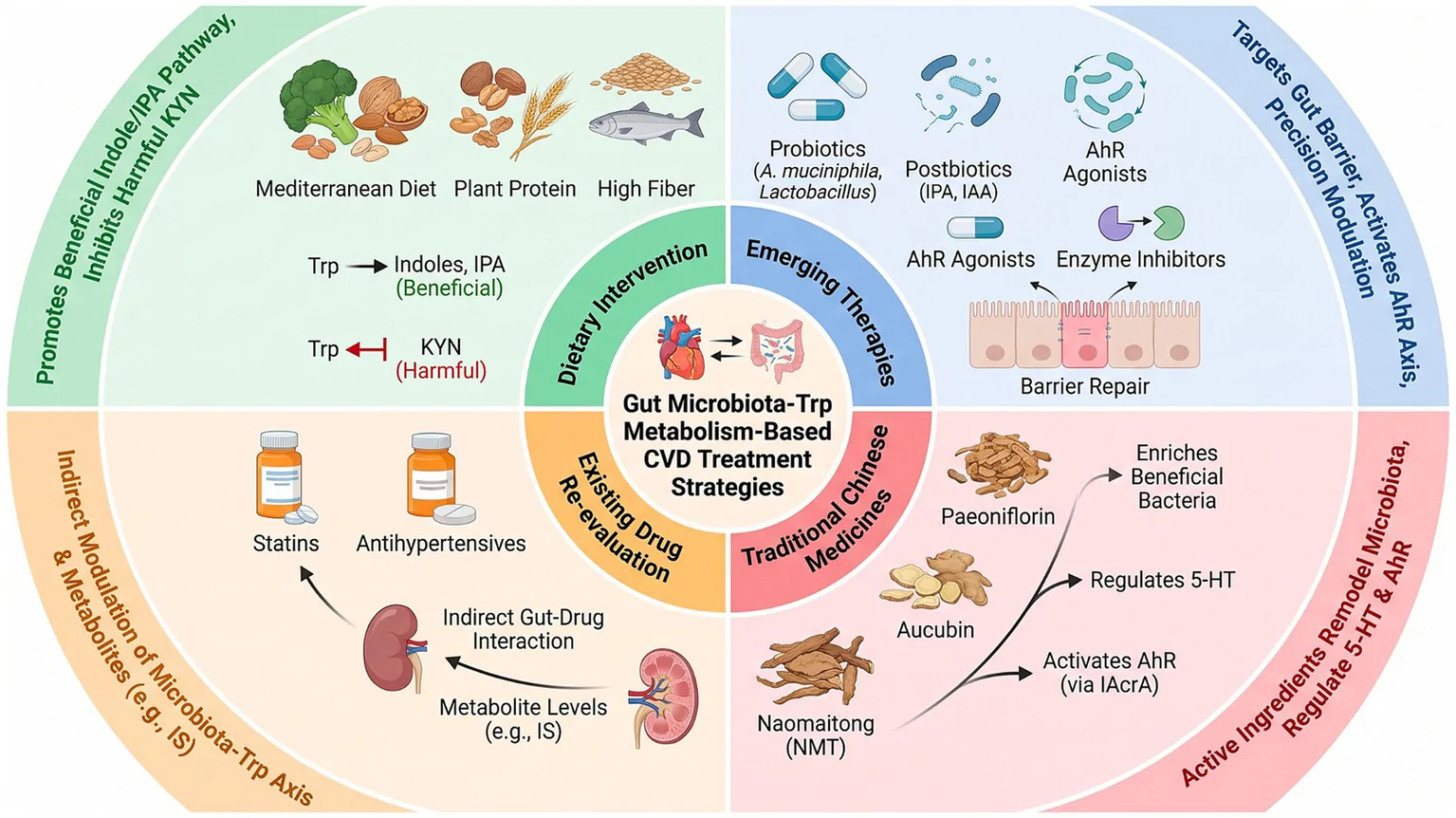
CVD treatment strategies based on gut microbiota-derived tryptophan metabolic pathways. This figure outlines four major categories of CVD intervention strategies targeting the gut microbiota Trp metabolic pathway. Dietary interventions regulate Trp metabolic flux through the Mediterranean diet, wheat bran, astaxanthin, mixed nuts, and plant-based protein diets, with the oral Trp load test providing a novel tool for personalized dietary guidance. Emerging therapies encompass modulation of the IDO1/5-HT pathway, supplementation with specific metabolites (IPA, IAA, IAcrA), AhR agonists, engineered probiotics (such as *Akkermansia muciniphila* and high-AhR-ligand-producing *Lactobacillus* strains), and precision interventions targeting PSRC1 genetic defects. Active ingredients of traditional Chinese medicine (paeoniflorin, Naomaitong, aucubin) exert anti-AS or neuroprotective effects by remodeling the gut microbiota-Trp metabolism axis. The Trp metabolism regulatory effects of existing cardiovascular drugs (e.g., dihydroquercetin) provide a new perspective for drug re-evaluation. Each strategy exerts anti-inflammatory, antioxidant, barrier repair, and cardiovascular function-improving effects by modulating microbial composition, metabolic enzyme activity, or key metabolite levels, ultimately through signaling pathways such as AhR, NRF2, and TGF-β/Smad, collectively forming a multi-layered, individualized CVD prevention and treatment system.

### 7.1 Dietary intervention

Dietary patterns exert differential effects on Trp metabolism and cardiovascular health depending on the baseline metabolic status of the population (Hussain et al., 2026). Rather than a uniform response, clinical outcomes vary significantly across healthy individuals, those with metabolic syndrome, and patients with established cardiovascular risk factors (Razquin et al., 2021; Wang et al., 2026).

In healthy and overweight populations, dietary interventions primarily aim to enhance metabolic flexibility and prevent inflammatory shifts. A randomized controlled trial (RCT) in 95 overweight/obese individuals demonstrated that mixed tree nut (MTN) intervention during a hypocaloric weight-loss phase specifically suppressed the pro-inflammatory Trp–KYN pathway, while the subsequent maintenance phase shifted flux toward the protective Trp–indole and Trp–5-HT pathways (Yang et al., 2023). Notably, this metabolic reprogramming was associated with tangible improvements in cardiovascular risk markers, such as reduced LDL cholesterol and improved insulin sensitivity, rather than just changes in microbiota diversity. The response was mediated by specific taxa, such as Paludicola, suggesting that individuals with lower baseline abundance of these bacteria may derive greater benefit from nut supplementation (Yang et al., 2023). Similarly, adherence to a Mediterranean diet in healthy cohorts has been linked to increased plasma levels of IPA and reduced kynurenine/tryptophan ratios, correlating with improved endothelial function and lower circulating IL-6 levels (Razquin et al., 2021).

In patients with T2D or high cardiovascular risk, the clinical focus shifts to reducing uremic toxins and systemic inflammation. Interventions with high-fiber components, such as bran or plant-based proteins, have shown distinct benefits in this subgroup (Yan et al., 2023). For instance, a crossover feeding trial in men with high cardiovascular risk revealed that a flexible vegetarian diet (64% plant protein) significantly reduced plasma levels of the uremic toxin IS and elevated circulating IPA (Lépine et al., 2026). Crucially, despite a reduction in overall indole-producing genetic potential in the gut microbiota, the shift toward IPA over IS highlights a qualitative improvement in metabolite profile that directly correlates with reduced endothelial dysfunction and oxidative stress markers in this high-risk group (Lépine et al., 2026). In contrast, Western diets in similar populations exacerbate the KYN pathway activation, leading to elevated kynurenine/tryptophan ratios, which are independently associated with increased incidence of MACE and higher hs-CRP levels (Hussain et al., 2026).

Personalized responses based on microbial phenotypes further define clinical efficacy. The oral Trp loading test has identified significant inter-individual variation in IS production among healthy subjects, driven by gut microbiota enzyme abundance rather than host genetics This stratification reveals that “high IS producers” represent a distinct subpopulation at elevated cardiovascular risk who may benefit disproportionately from low-Trp or high-fiber interventions, whereas “low IS producers” show minimal clinical gain from such restrictions (Lin T. Y. et al., 2025). This suggests that future dietary guidelines should move beyond one-size-fits-all recommendations to target specific microbial functional phenotypes.

In summary, the clinical effects of dietary interventions are not vague but are precisely modulated by baseline health status and microbial phenotype. Healthy individuals benefit from enhanced metabolic flexibility via indole/5-HT pathways, while high-risk patients achieve clinical protection through the suppression of uremic toxins (IS) and inflammatory kynurenines. Recognizing these population-specific responses is critical for designing effective precision nutrition strategies for CVD prevention.

### 7.2 Emerging therapies

In recent years, interventions targeting the gut microbiota and its derived Trp metabolites have gradually emerged as a new direction in the treatment of cardiovascular disease. In an Ldlr^−^/^−^ mouse model, intestinal IDO1 deficiency led to elevated 5-HT levels, disruption of the intestinal barrier and exacerbated atherosclerosis, while the use of 5-HT pathway inhibitors significantly increased fecal indole levels, improved intestinal barrier integrity, increased the expression of antimicrobial peptides and alleviated atherosclerosis, without altering plasma cholesterol levels, suggesting that the specific regulation of branch pathways of Trp metabolism holds therapeutic potential (Chajadine et al., 2024). IPA, a bacterial-derived Trp metabolite, has been shown in an *in vitro* cardiomyocyte model to alleviate doxorubicin-induced oxidative stress, inflammation and apoptosis in cardiomyocytes by inhibiting the HDAC6/NOX2 signaling pathway, demonstrating its potential in the treatment of heart failure (Li C. et al., 2024). Ganoderic acid A promotes the production of 3-IAld in a mouse model of colitis by modulating the gut microbiota, thereby activating AhR and inducing IL-22 production, which in turn repairs the intestinal barrier (Kou et al., 2024); *Akkermansia muciniphila* has also been shown in zebrafish models to enhance the production of ILA, indole-3-acetaldehyde and 5-hydroxyindoleacetic acid, thereby activating the AhR–IL-22 axis and alleviating high-fat diet-induced metabolic syndrome (Zhang F. et al., 2025). AhR agonists and specific Lactobacillus strains with high AhR ligand production capacity have also demonstrated therapeutic potential. Preclinical studies indicate that the ability of the gut microbiota to produce AhR ligands is significantly reduced in metabolic syndrome; whereas exogenous supplementation with AhR agonists or screened Lactobacillus strains with high AhR ligand production capacity can effectively alleviate diet-induced and genetically-related glucose metabolism abnormalities and hepatic steatosis by improving intestinal barrier function and promoting the secretion of the incretin glucagon-like peptide-1 (GLP-1); their efficacy has been validated in multiple models (Natividad et al., 2018). The above evidence suggests that targeting the Trp metabolic pathway through supplementation with probiotics, postbiotics, enzyme inhibitors or specific metabolites may provide a personalized and precision therapeutic approach for CVD.

Among intervention strategies targeting Trp metabolism, precision interventions addressing host genetic defects offer new prospects. In an atherosclerosis model using Psrc1-knockout mice, both oral supplementation with live *A. muciniphila* and direct supplementation with IAA significantly reversed plaque progression; the core protective mechanism lies in the inhibition of macrophage apoptosis within the plaques. Notably, these protective effects were completely abolished by the AhR-specific antagonist CH-223191, confirming that their efficacy is strictly dependent on the activation of the AhR pathway (Wu et al., 2026). This finding not only validates the feasibility of the “*A. muciniphila*–IAA–AhR” axis as a therapeutic target, but also suggests that for specific cardiovascular patients carrying loss-of-function variants of PSRC1, microbiome-targeted activation strategies based on this axis (such as supplementation with specific probiotics or postbiotics) hold promise as a personalized, precision treatment pathway; however, their clinical translation still requires validation through human trials.

The IDO1-kynurenine pathway has emerged as a druggable target, with several small-molecule inhibitors and inducers under development for cancer and other diseases. In the cardiovascular context, targeting specific components of this pathway—such as IDO1, kynurenine aminotransferase, or kynurenine 3-monooxygenase—could theoretically modulate the balance between beneficial and deleterious kynurenine metabolites. Furthermore, strategies that restore the balanced pool of host-derived and microbiota-derived AhR ligands may offer a novel therapeutic avenue for conditions where AhR dysfunction contributes to pathogenesis, such as atherosclerosis and ischaemic stroke (Russo et al., 2023). However, the context- and ligand-dependent nature of AhR activity poses a major limitation, and careful ligand selection and delivery will be essential to avoid unintended pro-inflammatory or cardiotoxic effects.

### 7.3 Treatment with traditional Chinese medicines

#### 7.3.1 Paeoniflorin

Paeoniflorin, as the primary active constituent of both white and red peony root, has been preliminarily suggested to exert a protective effect against atherosclerosis through the gut microbiota–Trp metabolism axis. Animal studies have shown that oral administration of paeoniflorin delays weight gain in ApoE^−^/^−^ mice fed a high-fat diet, reduces serum total cholesterol, triglycerides and LDL-C levels, alleviates aortic plaque formation, and downregulates TLR4 and NF-κB expression. At the mechanistic level, paeoniflorin can modulate linoleic acid metabolism, Trp metabolism and steroid biosynthesis pathways, while simultaneously improving abnormalities in galactose and glycerophospholipid metabolism, and restoring dysbiosis in the Firmicutes, Bacteroidetes and Actinobacteria phyla, thereby increasing the abundance of beneficial bacteria such as Lactobacillus and Bifidobacterium (Xia et al., 2025). These findings suggest that paeoniflorin may exert its anti-atherosclerotic effects by remodeling the microbiota–Trp metabolism axis; however, this conclusion is derived from a single ApoE^−^/^−^ mouse model, with no reported sample size or independent replication studies, and its clinical significance requires confirmation through human studies.

#### 7.3.2 Naomaitong

In the field of ischaemic stroke, the mechanism by which the traditional Chinese medicine Nao Miao Tong (NMT) promotes neurological recovery by modulating the gut microbiota–Trp metabolism axis has also been preliminarily explored. Animal studies have shown that NMT significantly reduces cerebral infarct volume, improves neurological function and restores intestinal barrier integrity in rats with a middle cerebral artery occlusion/reperfusion model. Metabolomic analysis revealed that NMT modulates the Trp pathway: 5-HT levels in the colon decrease, while 5-HT levels in plasma and cerebrospinal fluid increase. Concurrently, NMT enriches the abundance of *A. muciniphila* and regulates the expression of 5-HT-related enzymes (MAO-A, SERT, TPH1). Direct supplementation with *A. muciniphila* mimicked the protective effects of NMT, suggesting that it mediates dual brain–gut protection via microbiota–metabolite interactions (Liu B. et al., 2025). However, this study was also based on a single SD rat MCAO model and lacks independent validation and human data to support these findings.

#### 7.3.3 Aucubin

Aucubin is one of the major active constituents of *Eucommia ulmoides* Oliver; in recent years, preliminary evidence has suggested that its anti-atherosclerotic effects are closely related to the remodeling of Trp metabolic pathways in the gut microbiota. Animal studies have shown that oral administration of eucommioside significantly improves aortic plaque accumulation, inflammatory infiltration and endothelial function in ApoE^−^/^−^ mice, while increasing the thickness of the fibrous cap and reducing the necrotic core. Mechanism studies employing a combined strategy of fecal microbiota transplantation, 16S rRNA sequencing and non-targeted metabolomics revealed that the core mechanism of action of Aucubin lies in the selective enrichment of Lactobacillus species in the gut, thereby driving the metabolic conversion of Trp to indole-3-acrylic acid (IAcrA). This metabolite effectively inhibits endothelial-mesenchymal transition (EndMT) by activating the AhR receptor and blocking phosphorylation in the TGF-β/Smad pathway, thereby slowing the progression of atherosclerosis (Luo et al., 2024). This finding suggests that eucommioside may possess a novel “microbiota-dependent” pharmacological mechanism, and provides a potential molecular target for gut-targeted intervention strategies based on active constituents of traditional Chinese medicine; it also suggests that Lactobacillus abundance and circulating IAcrA levels may serve as potential biomarkers for assessing the efficacy of this class of traditional Chinese medicines. However, it should be noted that this conclusion is derived from a single ApoE^−^/^−^ mouse model, and no independent replicate experiments have been reported; its clinical translational value requires further toxicological evaluation and validation through human trials.

### 7.4 Regulatory effects of existing cardiovascular drugs on tryptophan metabolism

Although no cardiovascular drugs specifically targeting the gut microbiota’s Trp metabolic pathway have yet been approved, some existing drugs have been found to indirectly influence this metabolic axis. For example, dihydroquercetin alleviates systemic inflammation in a rat model of cerebral ischaemia-reperfusion injury by modulating Trp metabolism and gut dysbiosis; the mechanism involves changes in Trp metabolites mediated by the microbiota–gut–brain axis (Peng et al., 2025). Although research has primarily focused on models of neurological or metabolic diseases, the drug–microbiota–metabolism interaction mechanisms revealed by these studies offer a new perspective for the re-evaluation of cardiovascular drugs. It is worth noting that in patients with end-stage renal disease undergoing hemodialysis, gut microbiota is closely associated with levels of Trp metabolites (such as IS), and different metabolic pathways (such as alanine degradation and anaerobic fatty acid β-oxidation) are significantly correlated with metabolite abundance (Huang et al., 2024; Lin et al., 2022), suggesting that clinical medication or dialysis regimens may indirectly influence the state of Trp metabolism. Future research should systematically assess whether commonly used antihypertensive drugs, statins or anti-inflammatory agents confer additional benefits by modulating the gut microbiota and its Trp metabolites, thereby optimizing existing treatment strategies.

ST-120 (Kremezin), an oral spherical carbon adsorbent that non-specifically adsorbs indole and other uremic precursors in the gut, has been tested clinically as a means of lowering circulating IS levels. However, the two parallel phase 3 EPPIC trials (Evaluating Prevention of Progression In Chronic Kidney Disease; *n* = 2035) failed to demonstrate a significant effect of AST-120 (9 g/day) on the primary composite endpoint of time to dialysis initiation, kidney transplantation, or serum creatinine doubling in patients with moderate-to-severe CKD (Schulman et al., 2015). This negative outcome is crucial for a realistic appraisal of IS as a therapeutic target. It suggests that simply lowering circulating IS may not be sufficient to alter disease progression unless IS is a dominant driver in specific patient subgroups. These findings underscore the importance of patient stratification and the need to target upstream metabolic pathways or multiple metabolites simultaneously, rather than relying solely on a single scavenging strategy.

## 8 Limitations and future directions

### 8.1 Limitations

Although significant progress has been made in recent years regarding the role of gut microbiota-derived Trp metabolites in cardiovascular disease, current research still faces numerous limitations and unresolved issues.

A significant confounder in interpreting the clinical data on IS and related indolic metabolites, particularly in Sections 5.1 and 6, is their strong dependence on renal function (eGFR). The majority of the toxicological evidence for these compounds originates from patients with chronic kidney disease (CKD), where metabolite accumulation is pronounced. It remains unclear to what extent the observed associations between these metabolites and CVD in the general population are independent of eGFR, as many studies do not adequately adjust for this variable. Future prospective studies should incorporate rigorous eGFR adjustment to delineate the direct vascular toxicity of these metabolites from their role as markers of renal impairment. Beyond the issue of renal confounding, several other major limitations warrant consideration.

Firstly, most evidence originates from animal models or small-scale population cohorts, lacking support from large-scale prospective clinical studies. For example, although IPA has been shown to lower blood pressure in a hypertensive mouse model by regulating renal immune balance (Baranwal et al., 2023), the causal relationship and dose–response in human patients with hypertension remain unclear. Furthermore, conclusions regarding the effects of the same metabolite are contradictory across different studies: some studies suggest that IPA increases mean arterial pressure and enhances vasoconstriction (Konopelski et al., 2021), while others emphasize its cardioprotective effects by alleviating myocardial cell damage through inhibition of the HDAC6/NOX2 pathway (Li C. et al., 2024). This apparent contradiction likely reflects differences in experimental conditions rather than a true functional dichotomy. Pressor responses were observed only at suprapharmacological intravenous doses, whereas cardioprotective effects occur at physiological concentrations via oral administration (Baranwal et al., 2023; Wang et al., 2024). IPA activates distinct receptors in a tissue-specific manner: PXR in endothelium promotes vasodilation, while AhR in cardiomyocytes improves mitochondrial function (Wang Y. C. et al., 2024). Additionally, endothelial dysfunction in hypertension may unmask direct vasoconstrictive effects normally counterbalanced by endothelial mechanisms, and sex differences may further modulate these responses (Bardhan et al., 2024). Future studies using tissue-specific knockouts and physiologically relevant doses are needed to resolve these complexities. This “dual nature” of function suggests that the effects of Trp metabolites may be highly dependent on the disease context, the tissue microenvironment and receptor expression status.

Secondly, the complex mechanisms of interaction between the gut microbiota and host metabolism have not yet been fully elucidated. Existing studies have largely focused on individual bacterial species or single metabolites, while neglecting the overall functional synergy of the microbial community. For example, analysis of bacterial taxonomic units based on co-abundance clustering better reflects the association between gut microbiota and Trp metabolites than analysis of individual bacterial species (Sun et al., 2024; Hashikura et al., 2023), suggesting that focusing solely on individual “key bacteria” may fail to fully elucidate the metabolic regulatory network. Furthermore, the Trp metabolic pathway itself is highly branched, comprising the KYN pathway, the 5-HT pathway and the microbiota-dependent indole pathway; while there is cross-regulation between these pathways, systematic integrated analyses are currently lacking. Furthermore, certain metabolites, such as IAA, are positively correlated with mortality in patients with pneumonia (Kullberg et al., 2025), suggesting that their biological effects are context-dependent and may play distinctly different roles in various organ systems, which poses challenges for their application in the diagnosis and treatment of cardiovascular diseases.

Finally, existing technical methods still face bottlenecks in metabolite detection, gut microbiota functional annotation, and the dynamic monitoring of host–microbiota interactions. Although derivatisation-based LC–MS/MS methods can accurately detect a variety of Trp metabolites in plasma (van Faassen et al., 2019; Xu et al., 2026; Sadok and Staniszewska, 2021), their feasibility, cost-effectiveness and standardization for routine clinical application remain to be assessed. Furthermore, it is difficult to capture in real time the *in vivo* dynamic changes in gut microbiota enzyme activity (such as tryptophanase), which limits the accurate inference of metabolic flux.

### 8.2 Future directions

To overcome current research bottlenecks, future work should focus on the following areas: Firstly, large-scale, multicentre, longitudinal cohort studies should be conducted, combining metabolomics, metagenomics and clinical phenotypic data to systematically evaluate the dynamic changes in Trp metabolites across different subtypes of cardiovascular disease and their predictive value. Particular attention should be paid to the influence of confounding factors—such as gender, age, diet and medication use—on the metabolic profile, in order to enhance the specificity and generalisability of biomarkers. Secondly, the functional heterogeneity and mechanisms of action of Trp metabolites should be thoroughly elucidated. Conditional gene-knockout mice, organoid models and single-cell sequencing techniques should be utilized to identify receptor-dependent signaling pathways and their downstream effects of specific metabolites in different cell types. In particular, the molecular switches that determine whether the same metabolite exerts a protective or toxic effect under different pathological conditions need to be clarified. Thirdly, develop integrated multi-omics analysis strategies to construct a three-dimensional “microbiota–metabolism–host” interaction network. By integrating metagenomic, metatranscriptomic, metabolomic and host transcriptomic data, identify key functional microbiota modules driving Trp metabolism and the enzyme systems they encode; utilize synthetic biology approaches to reconstruct artificial microbiota and validate the causal relationship between their metabolic output and cardiovascular phenotypes. Fourthly, we will advance interventional research toward precision and personalisation. Based on an individual’s baseline microbiota structure and metabolic characteristics, we will design personalized dietary regimens, probiotic/prebiotic combinations or fecal microbiota transplantation protocols, and dynamically monitor the effects of these interventions on the remodeling of Trp metabolic pathways and their impact on endpoints such as blood pressure, atherosclerosis indices and cardiac function. Concurrently, explore the indirect regulatory effects of existing cardiovascular drugs on Trp metabolism to provide a basis for the repurposing of existing drugs. Fifth, develop high-throughput, non-invasive detection platforms. Optimize mass spectrometry and nuclear magnetic resonance (NMR) techniques to enable rapid screening of Trp metabolites in feces, urine or saliva; combine these with machine learning algorithms to construct diagnostic and prognostic prediction tools based on metabolite profiles, and facilitate their translation into clinical practice.

## 9 Conclusion

Tryptophan metabolites derived from the gut microbiota play a dual role in the onset and progression of cardiovascular diseases, exerting both protective effects and mediating pathological damage. Their core mechanisms involve multiple levels, including the maintenance of intestinal barrier integrity, the regulation of immune and inflammatory responses, and the modulation of neuroendocrine and blood pressure homeostasis. Current evidence suggests that these metabolites are not only potential novel biomarkers but also provide a theoretical basis for the development of intervention strategies based on the regulation of the gut microbiota. However, the complexity of their functions, limitations in research methods, and uncertainties regarding clinical translation continue to pose significant challenges. In the future, through interdisciplinary collaboration, technological innovation and rigorous clinical validation, it will be necessary to systematically elucidate the precise regulatory network of the Trp–microbiota–host axis in cardiovascular health, ultimately achieving a transition from mechanistic understanding to precision prevention and treatment.
